# Site-specific manipulation of *Arabidopsis* loci using CRISPR-Cas9 SunTag systems

**DOI:** 10.1038/s41467-019-08736-7

**Published:** 2019-02-13

**Authors:** Ashot Papikian, Wanlu Liu, Javier Gallego-Bartolomé, Steven E. Jacobsen

**Affiliations:** 1 Department of Molecular, Cell, and Developmental Biology, University of California, Los Angeles, CA 90095 USA; 2 Department of Human Genetics, David Geffen School of Medicine, University of California, Los Angeles, CA 90095 USA; 3 Molecular Biology Institute, University of California, Los Angeles, CA 90095 USA; 4 Howard Hughes Medical Institute, University of California, Los Angeles, CA 90095 USA

## Abstract

Understanding genomic functions requires site-specific manipulation of loci via efficient protein effector targeting systems. However, few approaches for targeted manipulation of the epigenome are available in plants. Here, we adapt the dCas9-SunTag system to engineer targeted gene activation and DNA methylation in *Arabidopsis*. We demonstrate that a dCas9-SunTag system utilizing the transcriptional activator VP64 drives robust and specific activation of several loci, including protein coding genes and transposable elements, in diverse chromatin contexts. In addition, we present a CRISPR-based methylation targeting system for plants, utilizing a SunTag system with the catalytic domain of the *Nicotiana tabacum* DRM methyltransferase, which efficiently targets DNA methylation to specific loci, including the *FWA* promoter, triggering a developmental phenotype, and the *SUPERMAN* promoter. These SunTag systems represent valuable tools for the site-specific manipulation of plant epigenomes.

## Abstract (web summary)

Few approaches for targeted manipulation of the epigenome are available in plants. Here, the authors adapt the dCas9-SunTag system to engineer targeted gene activation and site-specific manipulation of DNA methylation in *Arabidopsis*.

## Introduction

Gene transcription, and thus function, can be controlled in *trans*, via the binding of transcription factors to promoters, or in *cis*, via epigenetic modifications. Functional analyses of plant genomes have relied mainly on the indirect reactivation of genes or transposable elements (TEs) through the use of mutants^1^. However, the biological outcomes of these manipulations represent an aggregate view of gene expression changes. Examining epigenetic regulation of gene expression, such as by DNA methylation, faces similar issues, where indirect and widespread changes in epigenetic mutants complicate the exploration of locus-specific effects.

Mutant analysis, hairpin-mediated targeting of small interfering RNAs (siRNAs), and zinc-finger-mediated targeting have been used to assess the function of genes and DNA methylation in plants^1–3^. For example, fusion of a zinc-finger protein to the RNA-directed DNA methylation effector SUVH9 enabled methylation of the *Arabidopsis FWA* promoter^3^. The *fwa-4* (*fwa*) epiallele in *Arabidopsis* plants displays a loss of *FWA* promoter methylation, leading to *FWA* activation and a late flowering phenotype^3^. SUVH9-mediated de novo methylation of the *FWA* promoter in *fwa* plants restored *FWA* silencing and an early flowering phenotype^3^, indicating that promoter methylation was sufficient to regulate *FWA* expression. Although zinc-finger fusions are an effective tool, they are laborious to design, difficult to verify, and often display broad, off-target binding activity^4^.

CRISPR-Cas approaches enable targeted manipulation of specific loci^5^. Synthetic transcriptional activators, for instance consisting of deactivated versions of Cas9 (dCas9) fused to transcriptional activation domains, can specifically activate genes in both plants and mammals^6–12^. Several other CRISPR-Cas9-based activation systems, such as the synergistic activation mediator (SAM) as well as a hybrid VP64-p65-Rta (VPR) activator, have been developed to further enhance dCas9-mediated transcriptional upregulation as well as to recruit multiple protein effectors^13,14^. The dCas9-SunTag-VP64 system is a potent transcriptional activator in mammalian cell lines^15,16^. This system consists of two modules: dCas9 fused to tandem GCN4 peptide repeats, and a single chain variable fragment (scFv) GCN4 antibody fused to superfolder-GFP (sfGFP) and VP64. Thus, multiple copies of the VP64 transcriptional activator associate with the GCN4 repeats and are recruited to a specific locus via dCas9/guide RNAs. This method has been adapted for site-specific DNA demethylation in mammals and plants, and for DNA methylation in mammals^17–19^.

DNA methylation in plants exists in three different nucleotide contexts: CG, CHG, and CHH (where H = A, T, or C)^20^. Maintenance methylation is controlled by several pathways in *Arabidopsis*: CG methylation is maintained by DNA METHYLTRANSFERASE 1 (MET1), a homolog of DNMT1; CHG methylation is maintained by CHROMOMETHYLASE 3 (CMT3), a plant specific methyltransferase; and much of the CHH methylation is maintained by DOMAINS REARRANGED METHYLTRANSFERASE 2 (DRM2)-a homologue of DNMT3 methyltransferases-through the RNA-directed DNA methylation (RdDM) pathway. While DRM2 is responsible for CHH maintenance methylation in short euchromatic regions, short TEs, and the edges of long TEs, CHROMOMETHYLASE 2 (CMT2) is responsible for CHH methylation in pericentromeric heterochromatin and the bodies of long TEs^21,22^. RdDM is also responsible for de novo methylation of all three sequence contexts^20,23^.

Here, we have developed CRISPR-Cas9-SunTag-based targeting systems to site-specifically and efficiently manipulate gene methylation and expression in plants. We modified the SunTag system to recruit multiple copies of a methylation effector or of VP64 to distinct loci. Using the previously characterized *Nicotiana tabacum* DRM methyltransferase catalytic domain as our methylation effector^24^, we found that SunTag NtDRMcd effectively targets methylation to specific loci. Importantly, at the *FWA* locus, this methylated state remains meiotically heritable through multiple generations in the absence of the targeting transgene.

## Results

### Targeted transcriptional activation of the *FWA* locus

We previously adapted the SunTag system for site-specific DNA demethylation in plants by targeting the human TET1 catalytic domain to *Arabidopsis* loci^18^. To generate a transcriptional activator system, we used the *Arabidopsis UBIQUITIN10* (*UBQ10*) promoter to drive the expression of dCas9-10 × GCN4 and of scFv-sfGFP-VP64 (Supplementary Fig. 1a). Additionally, we added an SV40-type NLS to the scFv module to ensure proper nuclear import in plants. Imaging of the roots of T2 transgenic *Arabidopsis* plants expressing the SunTag VP64 construct showed clear nuclear localization of the antibody module (Supplementary Fig. 1b). In addition, dCas9-10 × GCN4 was stably expressed in T2 *Arabidopsis* plants (Supplementary Fig. 1c).

**Fig. 1:**
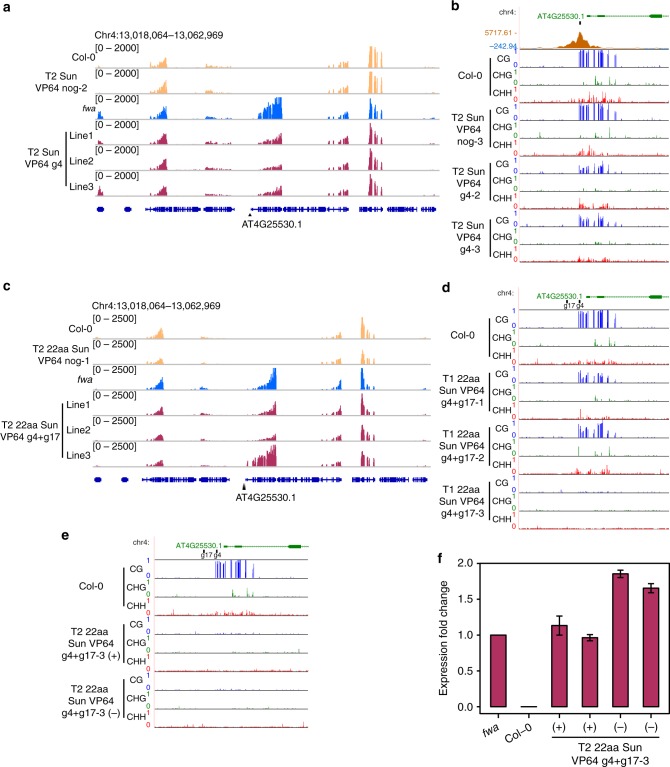
SunTag VP64-mediated *FWA* activation. **a** RNA-seq tracks depicting normalized reads at the *FWA* locus and flanking loci in 1 representative Col-0 replicate, 1 representative T2 SunTag VP64 nog-2 replicate, 1 representative *fwa* replicate, and 1 representative T2 SunTag VP64 g4 replicate for each of the 3 independent lines. The black triangle indicates the position of gRNA4. **b** ChIP-seq and WGBS tracks at the *FWA* promoter. The top track shows a ChIP peak corresponding to gRNA4-mediated SunTag recruitment. The position of gRNA4 is illustrated with a black bar. CG, CHG, and CHH methylation tracks for Col-0, T2 SunTag VP64 nog-3, and 2 independent T2 lines of SunTag VP64 g4. **c** RNA-seq tracks depicting normalized reads at the *FWA* locus and flanking loci in 1 representative Col-0 replicate, 1 representative T2 22aa SunTag VP64 nog-1 replicate, 1 representative *fwa* replicate, and 1 representative T2 22aa SunTag VP64 g4 + g17 replicate for each of the 3 independent lines. The black triangles indicate the positions of gRNA17 and gRNA4. **d** WGBS tracks for all 3 sequence contexts at the *FWA* promoter for Col-0 and 3 independent T1 lines of 22aa SunTag VP64 g4 + g17. The positions of gRNA17 and gRNA4 are illustrated with black bars. **e** WGBS tracks for all 3 sequence contexts at the *FWA* promoter for Col-0, and a T2 + and T2- plant from 22aa SunTag VP64 g4 + g17-3. **f** qRT-PCR analysis of *FWA* expression levels in *fwa*, Col-0, and 4 different T2 + or T2- plants from 22aa SunTag VP64 g4 + g17-3. Expression fold change relative to *fwa* is plotted. Error bars represent the mean ± s.e. of 2 technical replicates. Source data of Fig. 1f are provided as a Source Data file

To test whether this system activates gene expression, we targeted the DNA methylated and silent *FWA* gene in *Arabidopsis* wild-type (Col-0) plants^25^. We observed ectopic activation of *FWA* in numerous T1 lines containing a single guide RNA (gRNA4) that targets *FWA*, but not in control lines that lack a guide (nog) or that lack VP64 (Supplementary Fig. 2a, b). Strong activation of *FWA* was also observed in the next generation T2 plants (Supplementary Fig. 2b,c). RNA-seq of T2 gRNA4 plants confirmed that *FWA* was robustly upregulated (Fig. 1a and Supplementary Fig. 2d). In addition to gRNA4, we tested a guide (gRNA17) that targets a region further upstream in the promoter, ~170 base pairs upstream from gRNA4. We detected *FWA* upregulation with gRNA17, although to a lesser extent than with gRNA4, suggesting that gRNAs placed near the transcription start site may be more effective to manipulate gene expression, as previously suggested with the SunTag system in mammalian cell lines^16^ (Supplementary Fig. 2e).

To comprehensively profile the specificity of SunTag VP64-mediated activation, we examined differentially expressed genes (DEGs) in the gRNA4 RNA-seq dataset. All three profiled lines displayed highly specific activation of *FWA* with very few DEGs compared to a no guide control line (Supplementary Fig. 3a). To examine dCas9 binding at the *FWA* promoter, we performed ChIP-qPCR with T2 gRNA4 plants. We observed a strong enrichment of dCas9 at the *FWA* promoter compared to the control *ACT7* locus, and as expected, no enrichment in Col-0 control plants (Supplementary Fig. 3b). ChIP-seq showed highly specific binding of dCas9 to the *FWA* promoter, with only one major off-target site (Supplementary Fig. 3c). This off-target site contains a PAM and 14 base pairs complementary to the gRNA sequence, spanning the previously reported seed region of the protospacer^26^. Therefore, SunTag VP64-mediated gene activation is highly specific due to the highly specific binding properties of the Cas9/gRNA complex.

To test whether VP64-mediated *FWA* activation affected promoter methylation, we performed whole-genome bisulfite sequencing (WGBS) of T2 gRNA4 plants. Compared to Col-0 and no guide controls, T2 gRNA4 lines showed reduced CG methylation within the promoter, whereas gene body methylation downstream of the target site, as well as genome-wide methylation levels, remained unaffected (Fig. 1b and Supplementary Fig. 4a, b, c). Thus, targeted activation of silenced genes can reduce promoter methylation.

Although T2 gRNA4 plants showed *FWA* upregulation, they did not display altered flowering time compared to controls. Therefore, we hypothesized that the levels of *FWA* mRNA in T2 gRNA4 plants might not be sufficient to affect flowering time. In order to increase *FWA* expression even further, we combined gRNA4 and gRNA17 (g4 + g17) in one construct to test whether targeting two regions with gRNAs we have verified proximal to the promoter might further enhance activation. The 10 × GCN4 epitopes within the SunTag construct are separated by linkers of 5 amino acids (aa). To allow for maximum mobility of VP64, we also utilized 22aa linkers, as previously reported^17^. Furthermore, we added another SV40-type NLS within the coding sequence of dCas9-10 × GCN4. qRT-PCR analysis of T1 plants indicated that multiple lines with gRNA4 + gRNA17 had *FWA* expression levels similar to *fwa* epiallele plants, resulting in a late flowering phenotype (Supplementary Fig. 5a). RNA-seq in the T2 generation confirmed the upregulation of *FWA* expression, where, consistent with the qRT-PCR data, *FWA* transcript levels in line 3 were similar to those observed in *fwa* plants (Fig. 1c and Supplementary Fig. 5b). Analysis of DEGs showed that as with gRNA4, constructs with gRNA4 + gRNA17 were highly specific in activating *FWA*, with few other genes affected (Supplementary Fig. 5c).

We next tested how *FWA* promoter methylation was affected after activation with gRNA4 + gRNA17. We performed WGBS of T1 plants and observed a reduction of methylation in lines 1 and 2. Furthermore, promoter methylation in line 3 was completely abolished, correlating with expression data where *FWA* overexpression was similar to levels observed in *fwa* plants (Fig. 1c, d and Supplementary Fig. 5a, b). Thus, expression may be a key contributor that leads to this reduction in methylation. *FWA* gene body methylation remained unaffected in activated lines (Supplementary Fig. 6a). In the T2 generation, plants from line 3 that retained the transgene (+) and those that had it segregated away (−) both retained the demethylated state (Fig. 1e and Supplementary Fig. 6b). Consistently, we observed *FWA* expression levels similar to an *fwa* epiallele plant in both T2+ and T2− plants, correlating with the late flowering phenotype (Fig. 1f and Supplementary Fig. 6c).

### Activation of loci in different chromatin contexts

We next wanted to explore if other methylated loci, such as transposable elements (TEs), could be targeted for ectopic activation. We used two gRNAs to target *Evadé* (*EVD*), a member of the *ATCOPIA93* family of LTR/COPIA transposable elements^27^. Compared to control lines, 5aa SunTag VP64 T1 lines displayed retrotransposon activation by hundreds- to thousands of fold (Supplementary Fig. 7). We also confirmed the upregulation of *EVD* in three independent T2 lines (Supplementary Fig. 7).

**Fig. 2:**
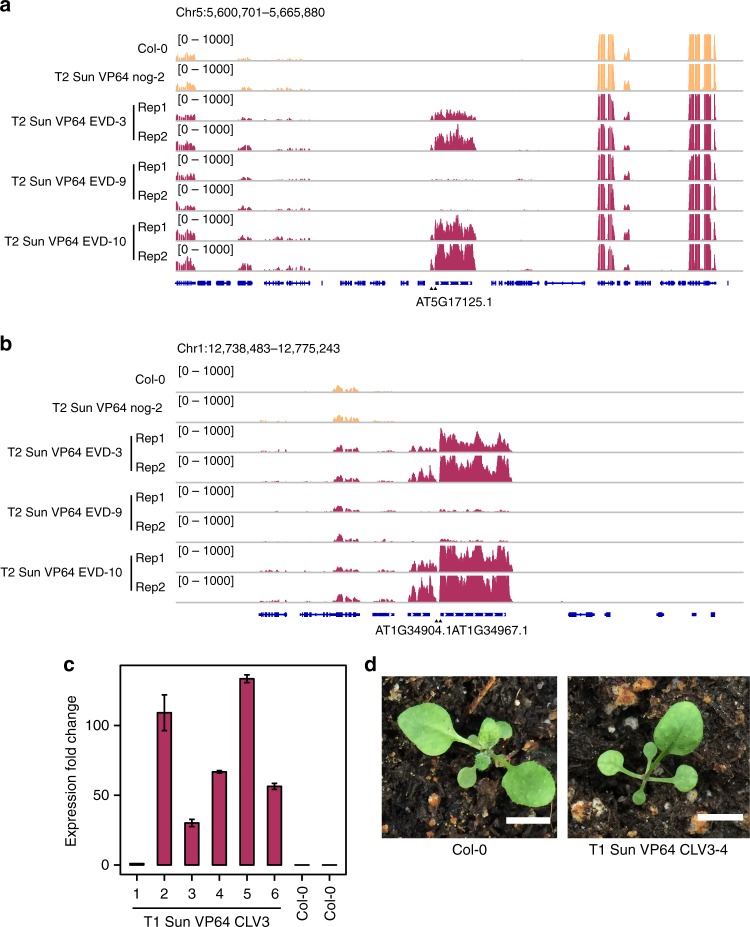
Activation of a diverse set of target loci. **a** RNA-seq tracks depicting normalized reads at the *EVD* locus and flanking loci in 1 representative Col-0 replicate, 1 representative T2 SunTag VP64 nog-2 replicate, and 2 representative T2 SunTag VP64 EVD replicates for each of the 3 independent lines. The black triangles indicate the positions of the 2 gRNAs. **b** RNA-seq tracks depicting normalized reads at the *ATR* locus and flanking loci in 1 representative Col-0 replicate, 1 representative T2 SunTag VP64 nog-2 replicate, and 2 representative T2 SunTag VP64 EVD replicates for each of the 3 independent lines. **c** qRT-PCR analysis of *CLV3* expression levels in 6 independent T1 lines of SunTag VP64 CLV3 and 2 independent Col-0 plants. Expression fold change relative to a line showing the lowest levels of upregulation (line 1) is plotted. Error bars represent the mean ± s.e. of 2 technical replicates. **d** A Col-0 plant and a representative T1 line of SunTag VP64 CLV3 is shown. Scale bars represent a distance of 5 mm. Source data of Fig. 2c are provided as a Source Data file

Next, we examined genome-wide effects by RNA-seq. The two gRNAs targeting the 5′ end of *EVD* had perfect matches to two different *ATCOPIA93* loci, one in euchromatin corresponding to the *EVD* locus, and another in heterochromatin corresponding to the *Attrapé* (*ATR*) locus. Both loci were highly activated, indicating that SunTag VP64 can manipulate gene expression in distinct chromatin contexts (Fig. 2a, b and Supplementary Fig. 8a, b). One neighboring TE of the same family adjacent to *ATR* was also upregulated (Fig. 2b). This effect might reflect co-regulation of these two TE copies and/or the presence of regulatory regions at the 3′ end. We observed robust activation of *EVD* and *ATR*, and few DEGs, in three independent T2 lines compared to a no guide control line (Supplementary Fig. 8c). Thus, SunTag VP64-mediated activation was highly specific.

We performed WGBS to monitor methylation levels proximal to the gRNA targets within *EVD* and *ATR*. We observed a decrease in CG methylation, while genome-wide methylation levels remained unaffected, recapitulating the effects observed at the *FWA* promoter (Supplementary Fig. 9a–d and 10). The SunTag system can thus be used to determine the biological consequences of activating specific TEs or TE families, rather than evaluating stress experiments or mutants that globally upregulate the expression of TEs^21,22,28,29^.

To test the generality of this activation system, we also targeted two unmethylated loci within the *Arabidopsis* genome. A zinc-finger-VP64 fusion has been shown to target and upregulate the expression of the floral development gene *APETALA3* (*AP3*)^30^. To test whether we could activate *AP3* with our system, we designed two gRNAs to target the *AP3* promoter. By qRT-PCR, we observed over 300-fold and 500-fold upregulation of *AP3* in T1 and T2 plants, respectively, compared to controls (Supplementary Fig. 11a). However, we did not observe the previously described *AP3* overexpression phenotype^30^, perhaps due to tissue-specific differences in transgene expression.

We also targeted the unmethylated stem cell regulator *CLAVATA3* (*CLV3*) with two gRNAs^31^. We observed strong upregulation of *CLV3* in several T1 lines where some exhibited the previously reported *wuschel* mutant phenotype-where the generation and maintenance of stem cells are perturbed in the meristem-arising from *CLV3* overexpression^31^, which persisted in T2 plants (Fig. 2c, d and Supplementary Fig. 11b). Thus, SunTag VP64 can site-specifically activate genes with methylated or unmethylated promoters.

### A SunTag-based methylation targeting system

Several reports have shown that dCas9 can be adapted to site-specifically modify DNA methylation in mammalian cells^19,32–36^. However, no such system yet exists to target DNA methylation in plants. We replaced VP64 in our SunTag system with the *Nicotiana tabacum* DRM methyltransferase catalytic domain (NtDRMcd) to evaluate methylation targeting activity in vivo. We chose NtDRMcd because this fragment was previously shown to be well-folded, well-expressed, and could be crystalized^24^. We utilized the *fwa* background, which has lost *FWA* promoter methylation, and established T1 plants that co-express gRNA4 to target the *FWA* promoter and 5aa SunTag NtDRMcd. These T1 plants showed some establishment of CHH methylation at the *FWA* promoter by WGBS, but very limited CG and CHG methylation (Supplementary Fig. 12a). The initial establishment of CHH methylation by NtDRMcd is consistent with the previously reported preference of tobacco DRM for non-CG methylation^37^. Ectopic methylation of the *FWA* promoter has been shown to generate early flowering plants^3^. However, we did not observe any flowering time differences between T1 plants and controls, likely due to the lack of CG methylation, which is required for *FWA* silencing^3^. Similarly, T2 plants showed slightly higher levels of CHH methylation but did not exhibit early flowering (Supplementary Fig. 12b).

To improve the efficiency of ectopic methylation targeting, we used our 22aa SunTag construct to accommodate optimum recruitment of NtDRMcd by mitigating the potential effects of steric hindrance. T1 22aa SunTag NtDRMcd gRNA4 transgenic lines showed enhanced *FWA* methylation compared to the 5aa T1 lines, including the establishment of CHH and CHG methylation, and minimal amounts of CG (Supplementary Fig. 12b). However, these plants also did not display the early flowering phenotype, suggesting that *FWA* expression was not silenced.

**Fig. 3:**
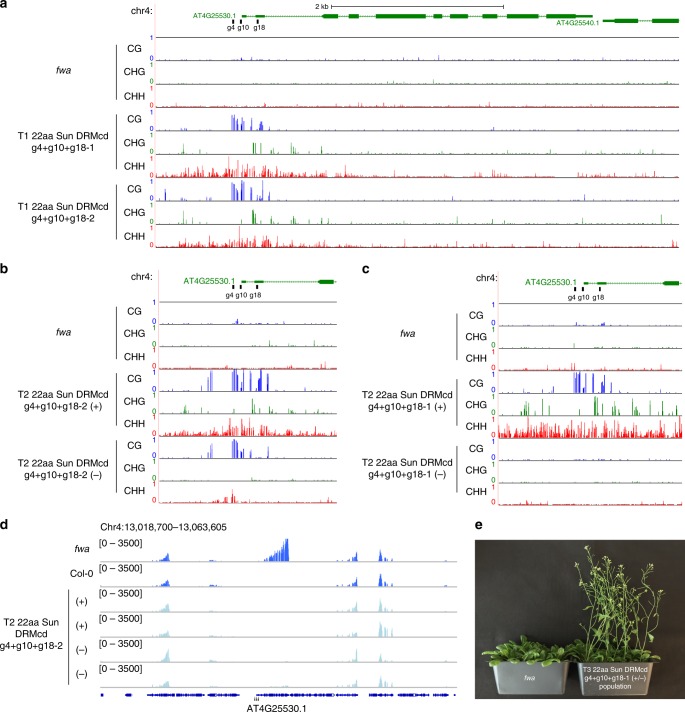
Targeted methylation and silencing of *FWA* in *fwa* epiallele plants. **a** WGBS tracks for all 3 sequence contexts are shown at the *FWA* locus for *fwa*, and 2 independent T1 lines of 22aa SunTag NtDRMcd g4 + g10 + g18. Black bars indicate the positions of the 3 gRNAs. **b** WGBS tracks for all 3 sequence contexts are shown at the *FWA* locus for *fwa* and T2 + and T2- plants from 22aa SunTag NtDRMcd g4 + g10 + g18-2. (+) and (−) indicate plants that have retained or segregated away the transgene, respectively. **c** WGBS tracks for all 3 sequence contexts are shown at the *FWA* locus for *fwa* and T2+ and T2− plants from 22aa SunTag NtDRMcd g4 + g10 + g18-1. **d** RNA-seq tracks depicting normalized reads at the *FWA* locus and its flanking regions in 1 representative *fwa* replicate, 1 representative Col-0 replicate, and 2 representative T2 22aa SunTag NtDRMcd g4 + g10 + g18 replicates each for T2+ and T2− plants. Black triangles indicate the positions of the 3 gRNAs. **e** Late flowering *fwa* plants are shown alongside a segregating population of T3 22aa SunTag NtDRMcd g4 + g10 + g18 plants that show the early flowering phenotype

To further enhance targeted methylation, we utilized two additional gRNAs targeting the *FWA* promoter. Together, these three gRNAs spanned the wild-type methylation patch observed in Col-0 plants. WGBS and McrBC analysis of T1 plants expressing 22aa SunTag NtDRMcd with gRNA4, gRNA10, and gRNA18 (g4 + g10 + g18) displayed efficient methylation establishment in all three sequence contexts within the *FWA* promoter (Fig. 3a and Supplementary Fig. 12c), which led to *FWA* silencing (Supplementary Fig. 13a) and early flowering plants. T2 plants that retained the SunTag NtDRMcd transgene (T2 + ) displayed *FWA* promoter methylation in both lines we followed (Fig. 3b, c). We identified T2 plants lacking the transgene (T2-) in line 2 that retained *FWA* promoter methylation, indicating that the targeted methylation was meiotically heritable (Fig. 3b). In line 1, *FWA* promoter methylation was lost in most T2- plants, which led to the reactivation of *FWA* (Fig. 3c and Supplementary Fig. 13a). Thus, the methylation established in T1 line 1 plants was insufficient to confer stable methylation and silencing in most T2- plants. In contrast, RNA-seq of line 2 T2 early flowering plants showed that *FWA* expression was silenced to wild-type levels in both T2+ and T2- plants, (Fig. 3d and Supplementary Fig. 13b), resulting in early flowering (Supplementary Fig. 13c).

We followed the T2 + early flowering plants of SunTag DRMcd g4 + g10 + g18 line 1 to the T3 generation. Both T3+ and T3- displayed robust *FWA* methylation and silencing (Supplementary Fig. 13d, e), as well as the early flowering phenotype (Fig. 3e). Thus, two generations in the presence of the SunTag NtDRMcd transgene were required for most plants to induce heritable methylation and silencing in this line. Furthermore, T4- plants derived from T3- plants continued to maintain *FWA* promoter methylation and the early flowering phenotype (Supplementary Fig. 13d, f). Although more independent events need to be characterized for further studies of methylation heritability, these findings are in line with the known intergenerational silencing effects associated with hairpins and at *FWA* and other loci^27,38–40^.

In addition to targeted DNA methylation, the scFv-sfGFP-NtDRMcd fusion induced background methylation throughout the genome, mainly in the CHH context, consistent with the reported preference of DRM^37^ (Supplementary Figs. 14 and 15a,b). We also observed chloroplast methylation in the presence of scFv-sfGFP-NtDRMcd (Supplementary Fig. 16a). Neither the genome-wide CHH hypermethylation nor the chloroplast methylation were retained in plants that had segregated away the transgene (Supplementary Figs. 15a, b, and 16a). Moreover, we profiled the methylation levels of *fwa* plants expressing the SunTag NtDRMcd transgene without a guide. T1 plants expressing this transgene showed no targeted methylation at *FWA* (Supplementary Fig. 16b), but again showed widespread background methylation that arises from the non-specific methyltransferase activity of NtDRMcd, as has been seen for targeting with mammalian methyltransferases^41^.

**Fig. 4:**
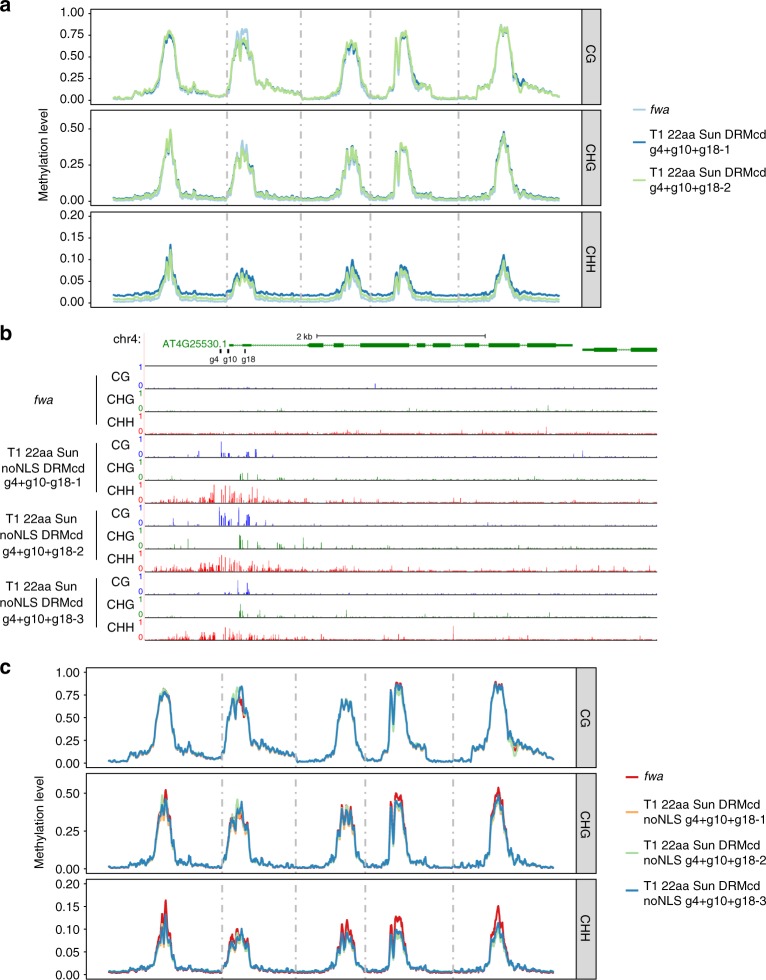
Profiling the genome-wide effects of scFv-sfGFP-NtDRMcd activity. **a** Chromosome-wide metaplots of CG, CHG, and CHH methylation levels in *fwa*, and 2 independent T1 lines of 22aa SunTag NtDRMcd g4 + g10 + g18. Dashed vertical lines depict the boundaries of chromosomes 1–5. **b** WGBS tracks for all 3 sequence contexts at the *FWA* promoter for *fwa* and 3 independent T1 lines of 22aa SunTag NtDRMcd noNLS g4 + g10 + g18. Black bars indicate the positions of the 3 gRNAs. **c** Chromosome-wide metaplots of CG, CHG, and CHH methylation levels in *fwa* and 3 independent T1 lines of 22aa SunTag NtDRMcd noNLS g4 + g10 + g18

To gain a better understanding of NtDRMcd off-target activity, we profiled genome-wide methylation levels in multiple generations of SunTag NtDRMcd g4 + g10 + g18 plants. Genome-wide methylation profiling of T1 plants showed hypermethylation throughout all chromosomes, mainly in the CHH context, with the CHG context being less affected and almost no effect on global CG methylation levels (Fig. 4a). In the T2 + generation, SunTag DRMcd g4 + g10 + g18 line 1 showed excessive hypermethylation in the CHH and CHG contexts. In contrast, T2 + line 2 showed minimal amounts of hypermethylation, and only in the CHH context (Supplementary Fig. 17a). These data emphasize that multiple lines should be evaluated in order to avoid those with excessive off-target hypermethylation. Importantly, segregating away the transgene reduced global hypermethylation to similar levels as the *fwa* control plants (Supplementary Fig. 17a). These findings were reiterated in T3+/− and T4− plants (Supplementary Fig. 17b).

We hypothesized that the SV40-type NLS in the scFv-sfGFP-NtDRMcd fusion may contribute to high levels of NtDRMcd in the nucleus leading to off-target DNA methylation. To test this hypothesis, we removed the SV40-type NLS, reasoning that nuclear localization of the scFv-sfGFP-NtDRMcd fusion would mainly occur only upon binding to the dCas9-NLS-10 × GCN4 fusion. Indeed, WGBS analysis of three independent T1 lines revealed that the SunTag NtDRMcd construct lacking the SV40-type NLS induced methylation at *FWA*, leading to early flowering, with limited effects on the surrounding regions (Fig. 4b and Supplementary Fig. 17c). Further, metaplots of genome-wide methylation showed that global CHH methylation levels in plants expressing the noNLS-SunTag NtDRMcd construct were similar to the *fwa* epiallele (Supplementary Fig. 17c and Fig. 4c). However, the noNLS-SunTag NtDRMcd construct was still able to access and methylate chloroplast DNA (Supplementary Fig. 18a). Thus, removal of the SV40-type NLS reduces nuclear off-target methylation of the SunTag NtDRMcd system.

**Fig. 5:**
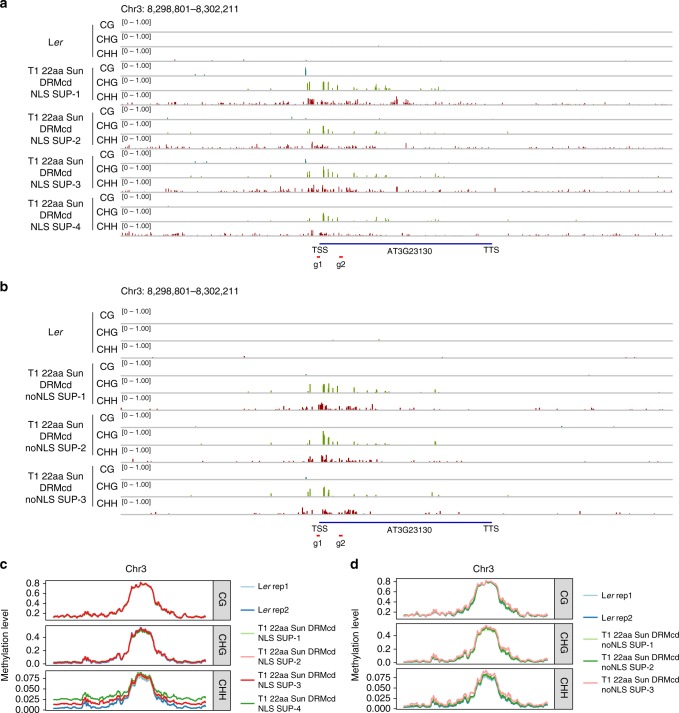
SunTag-mediated methylation targeting of the *SUPERMAN* locus. **a** WGBS tracks for all 3 sequence contexts are shown at the *SUP* locus for L*er* and 4 independent T1 lines of 22aa SunTag NtDRMcd NLS SUP. Red bars indicate the positions of the 2 gRNAs targeting *SUP*. TSS transcription start site, TTS transcription termination site. **b** WGBS tracks for all 3 sequence contexts are shown at the *SUP* locus for L*er* and 3 independent T1 lines of 22aa SunTag NtDRMcd noNLS SUP. **c** Chromosome 3 metaplots of CG, CHG, and CHH methylation levels in 2 different L*er* plants and 4 independent T1 lines of 22aa SunTag NtDRMcd NLS SUP. **d** Chromosome 3 metaplots of CG, CHG, and CHH methylation levels in 2 different L*er* plants and 3 independent T1 lines of 22aa SunTag NtDRMcd noNLS SUP

To test the generality of the SunTag NtDRMcd methylation targeting system, we used two gRNAs to target the floral development gene *SUPERMAN* (*SUP*) in the ecotype *Landsberg erecta* (L*er*)^42,43^. We profiled four independent T1 lines and observed efficient establishment of non-CG methylation (Fig. 5a), consistent with minimal CG sites in this region^42^. As with *FWA*, we observed off-target CHH methylation in the regions surrounding *SUPERMAN* (Supplementary Fig. 18b). We next utilized our noNLS version of the SunTag NtDRMcd construct to avoid genome-wide hypermethylation. Methylation was successfully targeted to the *SUPERMAN* promoter (Fig. 5b), with the surrounding regions now showing methylation profiles similar to the L*er* control (Supplementary Fig. 18c). Chromosome-wide metaplots showed that contrary to SunTag NtDRMcd, the noNLS version of the construct had global methylation levels similar to those observed in L*er* controls (Fig. 5c, d).

## Discussion

We have established SunTag systems to induce site-specific expression or methylation in *Arabidopsis*. SunTag VP64 is a highly efficient transcriptional activator that can be used to study the effects of overexpressing specific endogenous loci. In addition, conditional expression of VP64 could be used in the future to illuminate the tissue- and cell type-specific functions of genes. Compared to zinc fingers or TAL effectors fused to transcriptional activators, CRISPR-Cas9-based systems are more specific and efficient for multiplexing^44,45^, and simpler to engineer for new targets by simply designing new guide RNAs^5,46^. Because DNA methylated genes can be activated, this system provides an alternative to studying DNA methylation mutants, which can have indirect effects.

We also discovered an interesting phenomenon in which the promoter methylation of *FWA* was decreased or abolished as a result of activation, and also reduced at the 5′ end of *EVD* and *ATR* (Fig. 1b, d, e and Supplementary Fig. 9a–d). This observation suggests that gene expression plays an important role in reducing promoter methylation levels, through a mechanism that is either directly or indirectly perturbing methylation maintenance in promoter regions. Thus, gene activation and DNA methylation pathways are likely competing to regulate gene expression at promoters. Activation-mediated DNA demethylation further underscores that SunTag VP64 can be used to study the epigenetic regulation of methylated loci without altering global DNA methylation levels.

SunTag NtDRMcd-mediated DNA methylation targeting could be a valuable tool for agriculture for creating meiotically heritable epialleles without changing the DNA sequence^47^. As one example, plant genes required to support pathogenic bacteria or viruses could be targeted for promoter methylation and silencing^48^. Pleiotropic effects of silencing could potentially be avoided by fine-tuning the levels of repression caused by different levels of targeted methylation. Thus, the SunTag system is a versatile tool for site-specific manipulation of the epigenome in plants, and may have broad applications as a biotechnology tool.

## Methods

### Plant material and selection

The Columbia-0 (Col-0) ecotype of *Arabidopsis thaliana* was used in this study, along with the *fwa-4* epiallele, which was isolated from a *met1* segregating population^3^. The L*er* ecotype was used for targeting methylation to the *SUPERMAN* locus. To obtain transgenic lines, plants were transformed using the agrobacterium floral dip method^49^. Transgenic lines were obtained by selecting for hygromycin resistant plants on MS plates containing 0.9% Phytoagar (plantMedia), 1/2 Murashige and Skoog Basal Medium (MP Biomedicals, LLC), and 35 μg/mL Hygromycin B (Invitrogen). For microscopy, root tissue was used for obtaining confocal microscope images (Zeiss). Images were processed using Fiji^50^.

### SunTag cloning and design

Constructs used for activation and methylation targeting were cloned into either the pMOA binary vector^51^ or the pEG binary vector^52^. SunTag constructs were adapted from those described in Tanenbaum et al. in order to develop a plant specific SunTag system. Individual modules of the system in the constructs presented in this work were either PCR amplified from Addgene plasmid numbers 60903 and 60904 (gifts from Dr. Ron Vale, University of California, San Francisco) or were synthesized by GenScript.

The SunTag system consists of two main modules: dCas9 + epitope (10×GCN4) tail and a single chain antibody (scFv) fused to sfGFP and either VP64 or NtDRMcd. The epitope tail has a defined number of repeats separated by linker regions. In this study we employed 10 repeats of the GCN4 epitope. dCas9 + epitope tail expression was driven by the *Arabidopsis UBQ10* promoter that was followed by an omega translational enhancer. scFv module expression was also driven by the *UBQ10* promoter in the antisense orientation relative to *UBQ10*::dCas9 + epitope tail. gRNAs were cloned downstream from the scFv module and expressed using the U6 promoter. In order to express multiple gRNAs, each was cloned in tandem along with their independent U6 promoters. All cloning reactions were performed using In-Fusion (Takara) and all necessary components of the SunTag system were in a single binary vector.

In order to improve the DNA methylation targeting ability of the targeted NtDRMcd, we made a 22aa linker version of the dCas9 + epitope tail module in addition to the 5aa version, which represents the linker length in between epitope repeats. This was done to avoid the effects of steric hindrance since NtDRMcd is larger than the VP64 fusion. The 22aa linker version of dCas9 + epitope tail was also used for targeting VP64 to *FWA* with gRNA4 + gRNA17. In addition to adding this linker, we added an extra SV40-type NLS in between the 1xHA tag and the flexible linker separating dCas9 and the epitope tail. An SV40-type NLS was also added to the scFv module for effective nuclear import in *Arabidopsis*. Plasmids related to this study have been deposited to Addgene for the scientific community (Plasmid numbers: 115480-115490, 117168, 119554, 119672, and 120249–120252).

We designed numerous guides in the *FWA* promoter region. The designed guides were numerically numbered and specific ones were chosen for either activation or methylation targeting. The use of specific guides does not indicate that others did not work. For example the use of gRNA4 does not indicate that gRNAs1-3 did not work. It was chosen for its position in the *FWA* promoter.

### Western blotting

To extract total protein, leaf tissue was boiled at 95 °C with 2 × SDS buffer for 5 min. Raw protein extracts were then run with 3-8% Tris-acetate gels (NuPAGE) at 180-200 V. An iBlot gel transfer instrument (Thermo Fisher) was then used to transfer proteins to a PVDF membrane. Ponceau staining was used for visualizing loading controls. anti-HA-Peroxidase, High Affinity antibodies (Roche, catalog #12 013 819 001, clone BMG-3F10) were used for blotting.

### qRT-PCR analysis

For all qRT-PCR experiments, RNA was first extracted from leaf tissue using the Direct-zol RNA MiniPrep kit (Zymo). 500 ng–1 μg of total RNA was then used for cDNA synthesis using the SuperScript III First-Strand Synthesis Supermix (Invitrogen). qPCR analysis was then done using the iQ SYBR Green Supermix (Bio-Rad) to detect transcript expression levels. *FWA* transcripts were detected using oligos 5′-TTAGATCCAAAGGAGTATCAAAG-3′ and 5′-CTTTGGTACCAGCGGAGA-3′. *EVD* transcripts were detected using the previously reported oligos 5′-GATAGAGGAGATAGAAGATCTACAACTGG-3′ and 5′-CTCTATACTCCGATTCTGCACTCGAACA-3′^27^. *AP3* transcripts were detected using the previously reported oligos 5′-TTTGGACGAGCTTGACATTCAG-3′ and 5′-CGCGAACGAGTTTGAAAGTG-3′^30^. *CLV3* transcripts were detected using the previously reported oligos 5′-GTTCAAGGACTTTCCAACCGCAAGATGAT-3′ and 5′-CCTTCTCTGCTTCTCCATTTGCTCCAACC-3′^53^. All transcripts were normalized to the housekeeping gene *ISOPENTENYL PYROPHOSPHATE:DIMETHYLALLYL PYROPHOSPHATE ISOMERASE 2* (*IPP2*). *IPP2* transcripts were detected using oligos 5′-GTATGAGTTGCTTCTCCAGCAAAG-3′ and 5′-GAGGATGGCTGCAACAAGTGT-3′.

### McrBC assay

To detect differences in methylation using the endonuclease McrBC (NEB), genomic DNA was first extracted using either a CTAB-based method or the DNeasy Plant Mini Kit (Qiagen). 100 ng of genomic DNA was then digested for 4 h at 37 °C. Water was added instead of McrBC for undigested control samples. After digestion, qPCR analysis was done using the iQ SYBR Green Supermix (Bio-Rad) to quantify differences in methylation. *FWA* promoter region sequences were detected using oligos 5′-TTGGGTTTAGTGTTTACTTG-3′ and 5′-GAATGTTGAATGGGATAAGGTA-3′.

### RNA-seq library preparation and analysis

For transcriptome analyses, RNA was first extracted from leaf tissue using the Direct-zol RNA MiniPrep kit (Zymo). 1 μg of total RNA was used to prepare libraries for sequencing using the TruSeq Stranded mRNA-seq kit (Illumina). Libraries for SunTag VP64 targeting *EVD* were prepared using the TruSeq Stranded Total RNA with Ribo-Zero kit (Illumina). For sequencing of SunTag VP64 targeting *FWA* and SunTag NtDRMcd targeting *FWA*, we obtained single-end 50 bp reads. For sequencing of SunTag VP64 targeting *EVD*, we selected for larger fragment sizes and obtained paired-end 100 bp reads. Sequencing reads were first aligned to the TAIR10 gene annotation dataset using TopHat (2 mismatches allowed)^54^. We used the one max multihits option (-g 1) and --no-coverage-search. If reads did not map to genes, they were aligned to the TAIR10 genome. Read counts were obtained by implementing HTSeq^55^ and subsequent differential expression analyses were done using DESeq (Bioconductor) and custom R scripts.

### ChIP

T2 SunTag VP64 gRNA4 and Col-0 control plants were first grown on MS plates for 2 weeks and 2 grams of tissue were then collected per sample. After grinding the tissue, samples were crosslinked in 1% formaldehyde, chromatin was extracted, and later sonicated using Bioruptor Plus (diagenode). Immunoprecipitations were performed using mouse monoclonal anti-HA.11 epitope tag antibodies (clone 16B12, Covance catalog #MMS-101R). Chromatin-protein complexes were isolated with a 1:1 mix of Protein A and Protein G Dynabeads (Invitrogen) for 3 h at 4 °C. Beads were washed with low salt buffer (2 × ), high salt buffer, LiCl buffer, and TE buffer, and complexes were eluted with elution buffer (2 × 20 min at 65 °C). DNA-protein complexes were reversed crosslinked overnight at 65 °C followed by proteinase K treatment at 45 °C for 5 h. DNA was purified using phenol:chloroform, followed by NaOAc/EtOH precipitation along with GlycoBlue (Invitrogen) overnight at -20 °C. DNA was washed with 70% EtOH and resuspended with water. For ChIP-qPCR, the *ACT7* locus was detected using the oligos 5′-AGCACGGATCGAATCACATA-3′ and 5′-CTCGCTGCTTCTCGAATCTT-3′. For detection of the *FWA* locus, oligos 5′-AAGAGTTATGGGCCGAAGC-3′ and 5′-CGCTCGTATGAATGTTGAATG-3′ were used. Libraries were prepared using the Ovation Ultralow kit (NuGen). ChIP-seq analysis was done by uniquely aligning single-end 50 bp reads to the TAIR10 genome using Bowtie^56^ allowing two mismatches (-v 2). Subsequently, peaks were called using MACS2^57^ with default parameters. We identified 3 peaks, including *FWA*, at FDR 5% and above five-fold enrichment. An off-target peak from within this set of 3 peaks was defined by the presence of a potential gRNA binding site in proximity to a called MACS2 peak. We identified one major off-target peak for gRNA4 on chromosome 4.

### WGBS library preparation and analysis

For the preparation of WGBS libraries, genomic DNA was first extracted from leaves and inflorescence tissue (for L*er* samples) using the DNeasy Plant Mini Kit (Qiagen). 100 ng of DNA was then used for subsequent shearing using a Covaris S2 Focused Ultrasonicator. Libraries were then prepared using either the Ovation Ultralow Methyl-Seq kit (NuGen) in conjuction with the EpiTect Bisulfite Kit (Qiagen), or the Hyper Prep Kit (KAPA Biosystems) in conjuction with either the EZ DNA Methylation-Lightning Kit (Zymo) or the EpiTect Bisulfite Kit (Qiagen). Single-end 50 bp reads were then uniquely aligned to the TAIR10 genome using BS-Seeker2^58^. Methylation levels were then calculated for the CG, CHG, and CHH contexts. A filter was implemented to remove reads with three or more consecutively methylated cytosines in the CHH context, as previously described^59^. Metaplots of BS-seq data were generated with custom Python and R scripts. For methylation calculations over individual chromosomes, each chromosome was split into 100 kb bins. Methylation values were then calculated from these bins.

### Code availability

Custom code/scripts used in this study are available upon request. Custom code/scripts used in this study for generating methylation metaplots have been deposited on GitHub (https://github.com/wanluliu/WGBS_chromosome_metaplot).

### Reporting summary

Further information on experimental design is available in the Nature Research Reporting Summary linked to this article.

## Supplementary information

Supplementary Information

Reporting Summary

Source Data

## Data Availability

The data supporting the findings of this study are available within the article and its Supplementary Information. High throughput sequencing data has been deposited in the Gene Expression Omnibus (GEO) database and can be accessed with the accession number GSE125230. The source data of Figs. 1f and 2c, and Supplementary Figs. 1c, 2a, 2b, 2c, 2e, 5a, 7, 11a, 12c, 13a, and 13e are provided as a Source Data file. All data are available from the corresponding author upon reasonable request. A reporting summary for this Article is available as a Supplementary Information file.
