# Supplementary material for: Site-specific manipulation of Arabidopsis loci using CRISPR-Cas9 SunTag systems
Source: Nat Commun. 2019 Feb 13;10:729. doi: 10.1038/s41467-019-08736-7 (PMC6374409; doi:10.1038/s41467-019-08736-7)
Supplement: Supplementary file 1 — Supplementary Information [file 41467_2019_8736_MOESM1_ESM.pdf]

Supplementary Information

Site-specific manipulation of *Arabidopsis* loci using CRISPR-Cas9 SunTag systems

Papikian et al.

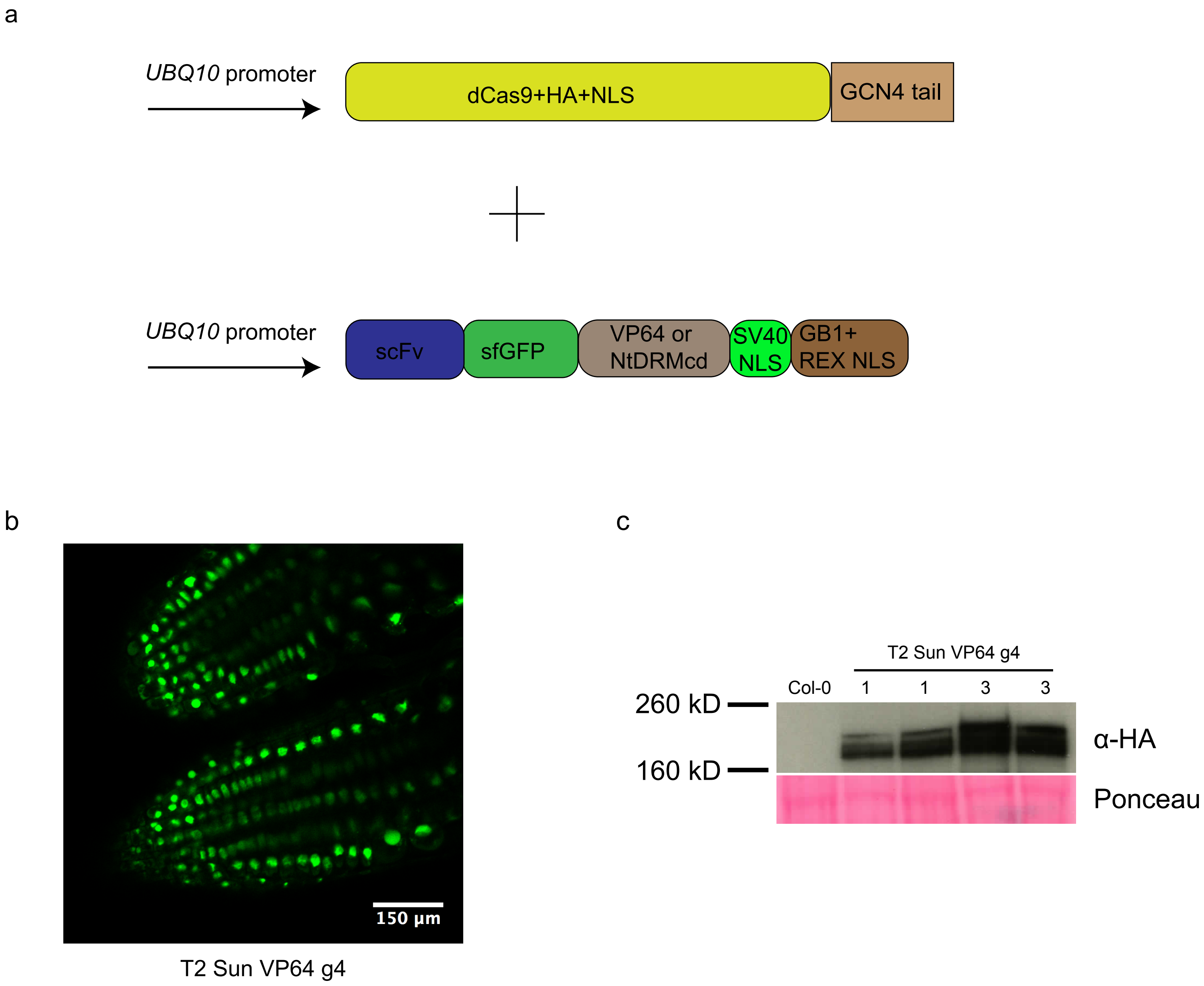

Supplementary Figure 1 The SunTag system is expressed in plants. (**a**) Schematic illustrating the two modules of the SunTag construct consisting of dCas9-10×GCN4 epitope tail whose expression is controlled by the *UBQ10* promoter, and scFv-sfGFP-(VP64 or NtDRMcd)-GB1-REX NLS (NLS present in the SunTag construct for mammals, as reported in Tanenbaum et al., 2014), whose expression is also controlled by the *UBQ10* promoter. (**b**) Confocal microscope image of *Arabidopsis* roots of T2 plants showing GFP fluorescence as a result of scFv-sfGFP-VP64 expression. An SV40-type NLS was also added to the scFv fusion. (**c**) Western blot analysis of dCas9-10×GCN4 expression levels in a wild type Col-0 plant and 2 independent T1 lines (2 biological replicates each) of SunTag VP64 gRNA4 (g4). Ponceau staining was used for visualizing loading controls. A horseradish peroxidase coupled anti-HA antibody was used to detect dCas9-10×GCN4 (~190kD). Source data of Supplementary Figure 1c are provided as a Source Data file.

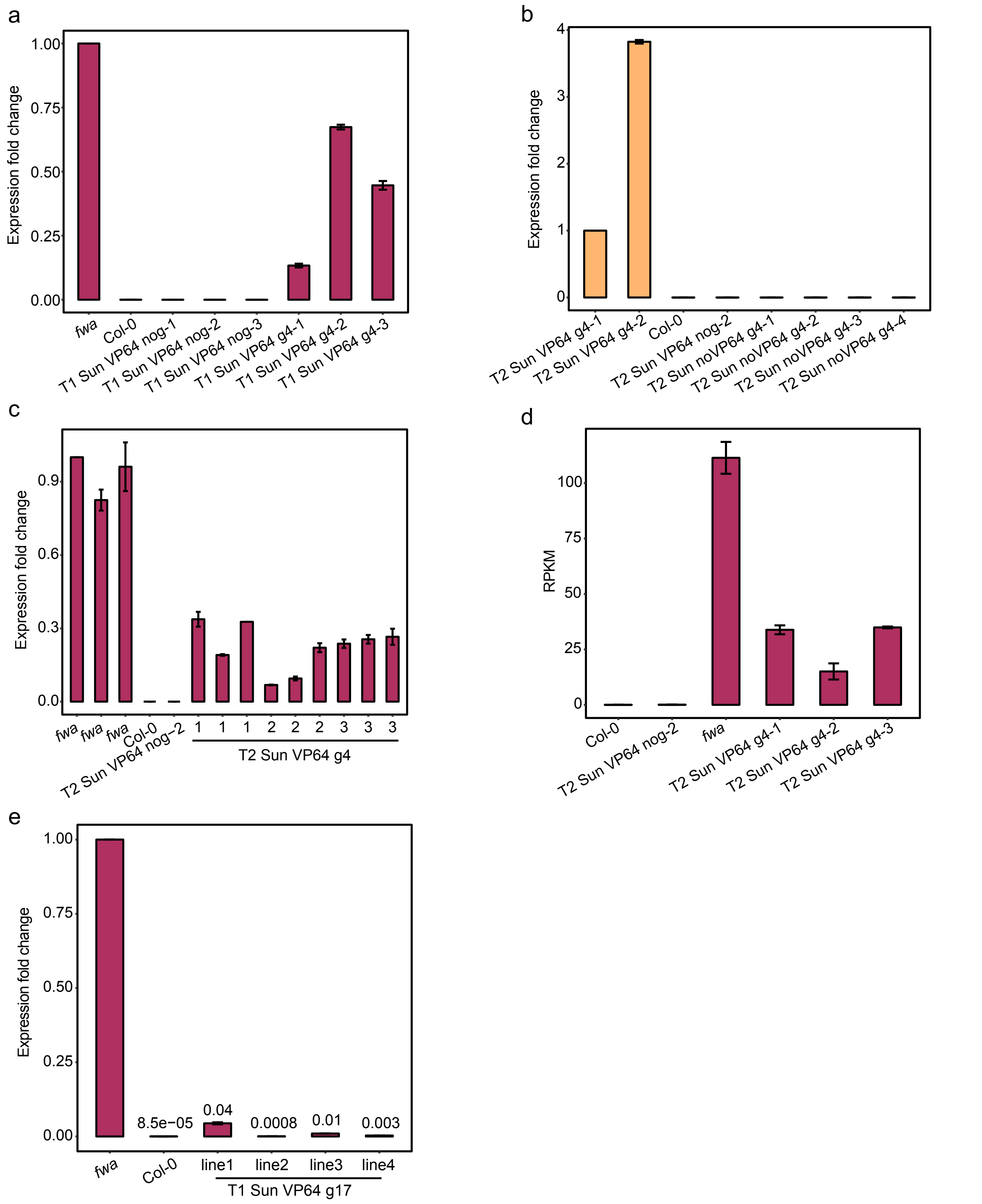

Supplementary Figure 2 Characterizing SunTag VP64-mediated *FWA* activation. (**a**) qRT-PCR analysis of *FWA* expression levels in *fwa*, Col-0, 3 independent T1 SunTag VP64 nog lines, and 3 independent T1 SunTag VP64 g4 lines. Expression fold change relative to *fwa* is plotted. Error bars represent the mean ± s.e. of 2 technical replicates. (**b**) qRT-PCR analysis of *FWA* expression levels in 2 independent T2 lines of SunTag VP64 g4, Col-0, T2 line of SunTag VP64 nog-2, and 4 independent T2 lines of SunTag noVP64 g4. Expression fold change relative to T2 SunTag VP64 g4-1 is plotted. (**c**) qRT-PCR analysis of *FWA* expression levels in 3 different *fwa* plants, Col-0, T2 SunTag VP64 nog-2, and 9 different plants of T2 SunTag VP64 g4 from 3 independent lines. Expression fold change relative to the first *fwa* plant is plotted. (**d**) RPKM values from RNA-seq data of Col-0, T2 SunTag VP64 nog-2, *fwa*, and 3 independent T2 lines of SunTag VP64 g4. Error bars represent the mean ± s.e. of biological replicates: 3 for Col-0, 4 for T2 SunTag VP64 nog-2, 3 for *fwa*, 4 for T2 SunTag VP64 g4-1, 3 for g4-2, and 4 for g4-3. (**e**) qRT-PCR analysis of *FWA* expression levels in *fwa*, Col-0, and 4 independent T1 lines of SunTag VP64 g17. Expression fold change relative to *fwa* is plotted. Source data of Supplementary Figures 2a,b,c, and e are provided as a Source Data file.

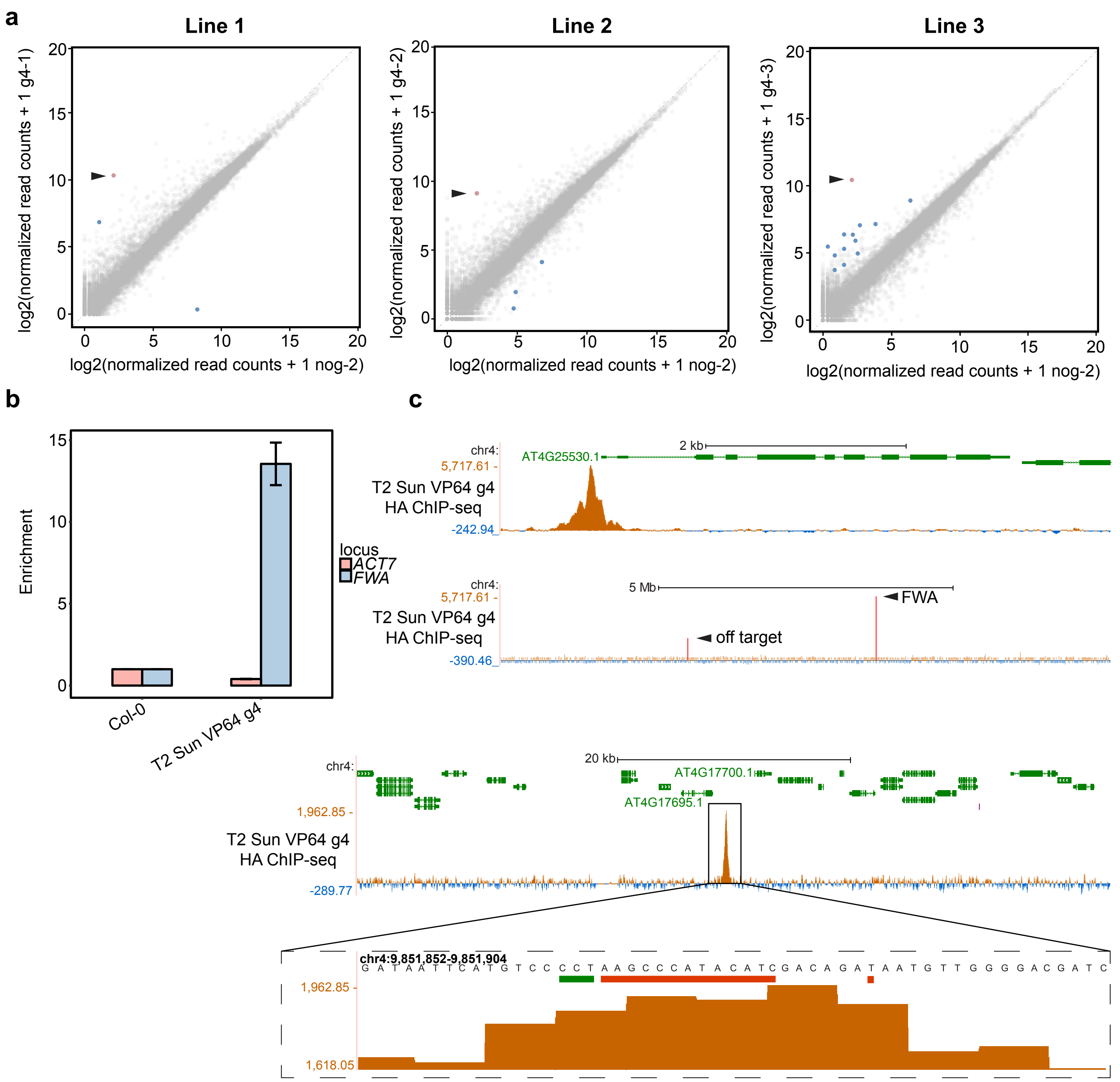

Supplementary Figure 3 SunTag VP64 activation is highly specific. (**a**) Scatterplots showing the DEGs of T2 SunTag VP64 g4-1, g4-2, and g4-3 plants versus SunTag VP64 nog-2 plants. Gray dots represent non-DEGs and blue dots represent DEGs. The red dot with a black triangle represents the differential expression of *FWA*. (**b**) ChIP-qPCR data from SunTag VP64 g4 HA ChIP. The enrichment over *ACT7* and the *FWA* promoter in T2 SunTag VP64 g4 plants relative to Col-0 is plotted. Error bars represent ± s.d of 3 technical replicates. (**c**) ChIP-seq tracks of SunTag VP64 g4 normalized to a Col-0 control. The top panel shows a zoomed in view of the ChIP peak at the *FWA* promoter, the middle panel shows tracks from a portion of chromosome 4 showing the *FWA* peak and the only genome-wide major off target, and the bottom panel shows the major off target peak for gRNA4. A zoom-in is shown where a green bar indicates the presence of a PAM sequence and red bars indicate bases complementary to gRNA4.

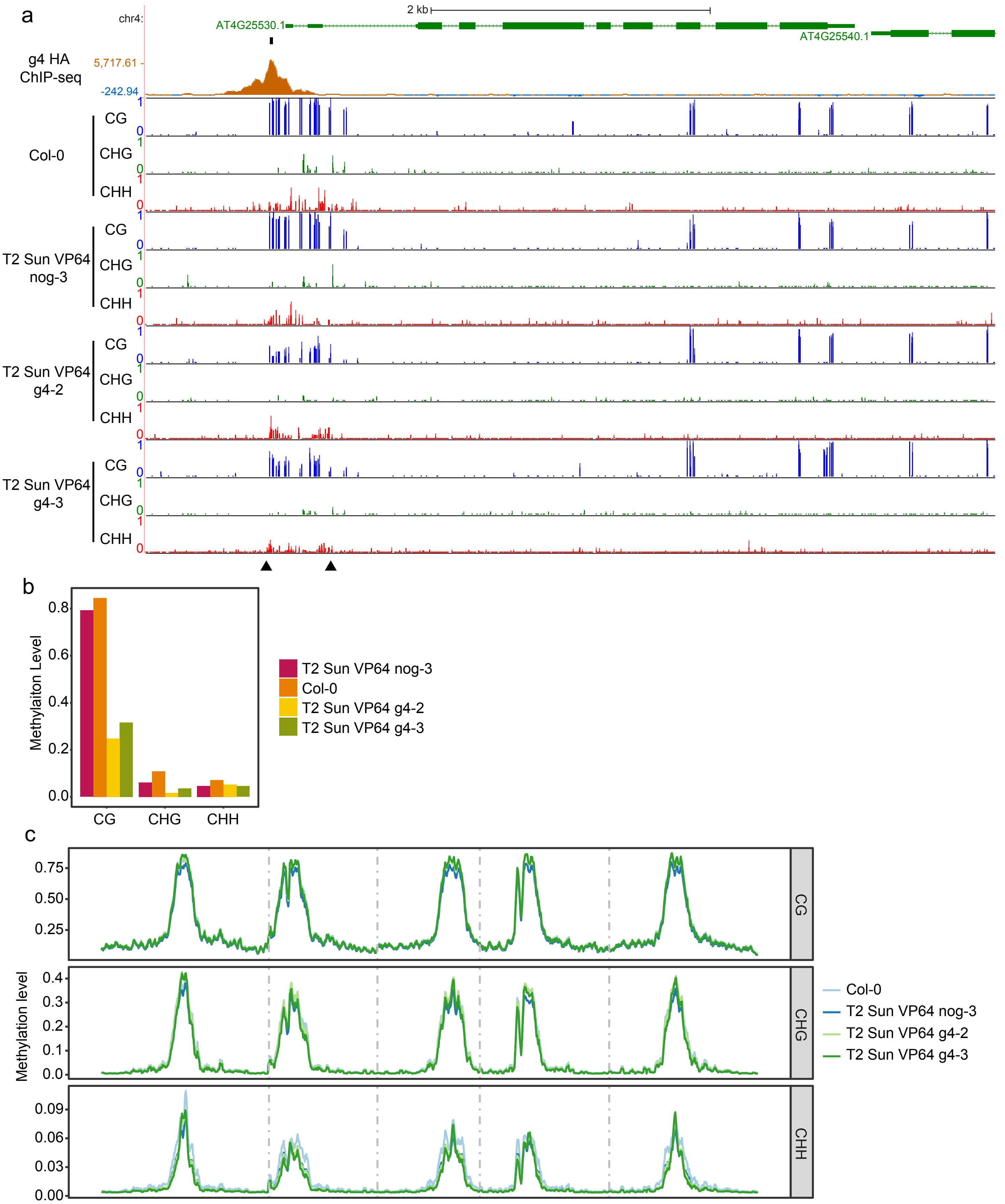

Supplementary Figure 4 *FWA* activation leads to a decrease in methylation at the *FWA* promoter. (**a**) ChIP-seq and WGBS tracks at the *FWA* genomic region. The top track shows a ChIP peak corresponding to gRNA4-mediated SunTag recruitment. The position of gRNA4 is illustrated with a black bar. CG, CHG, and CHH methylation tracks for Col-0, T2 SunTag VP64 nog-3, and 2 independent T2 lines of SunTag VP64 g4. (**b**) Barplot showing CG, CHG, and CHH methylation levels in T2 SunTag VP64 nog-3, Col-0, and 2 independent T2 lines of SunTag VP64 g4. Black triangles in **a** indicate the boundaries used to calculate the methylation levels in the *FWA* promoter region. (**c**) Chromosome-wide metaplots of CG, CHG, and CHH methylation levels of Col-0, T2 SunTag VP64 nog-3, and 2 independent T2 lines of SunTag VP64 g4. Dashed vertical lines depict the boundaries of chromosomes 1-5.

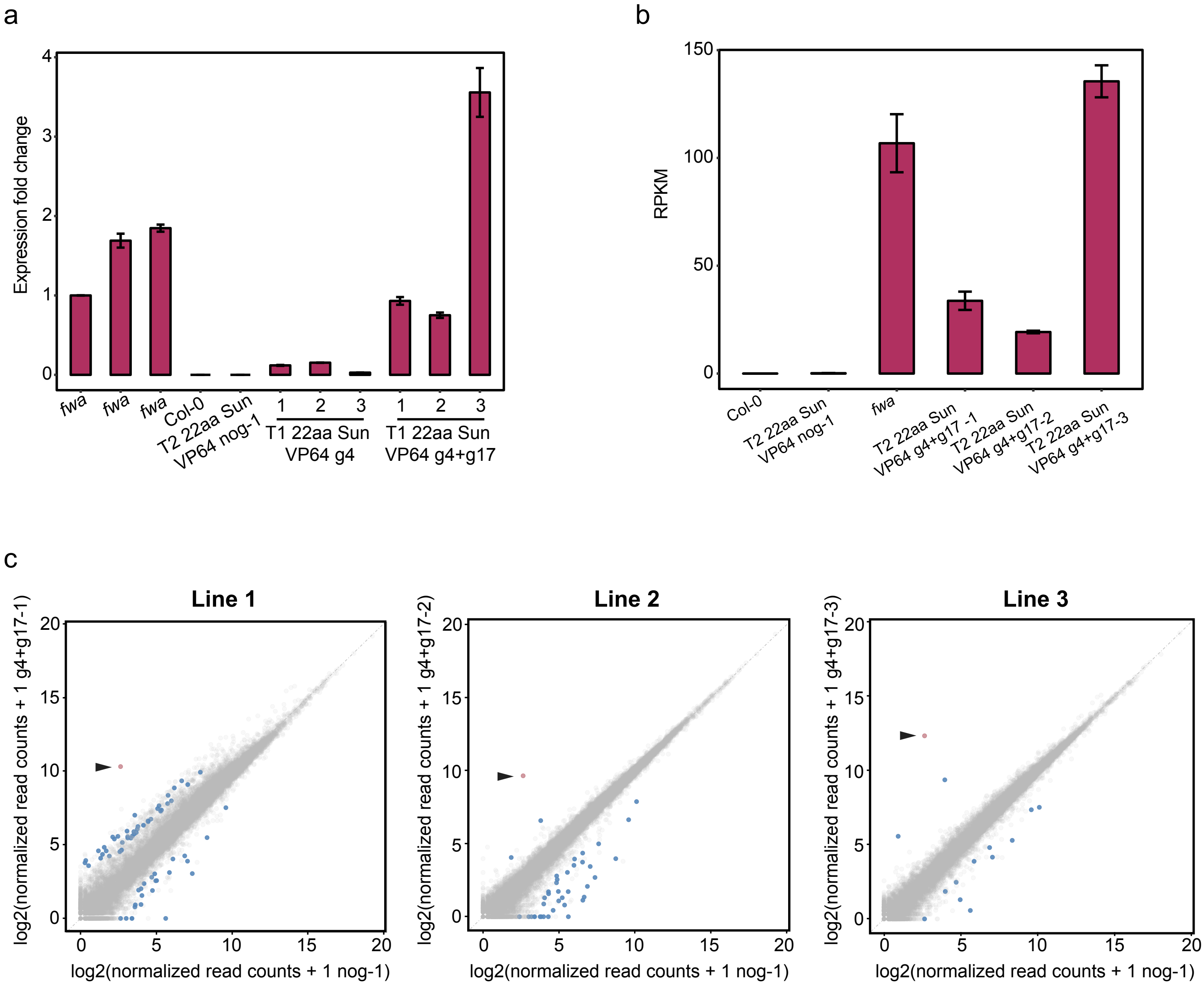

Supplementary Figure 5 Enhanced and specific activation of *FWA* using two gRNAs. (**a**) qRT-PCR analysis of *FWA* expression levels in 3 different *fwa* plants, Col-0, T2 22aa SunTag VP64 nog-1, 3 independent T1 lines of 22aa SunTag VP64 g4, and 3 independent T1 lines of 22aa SunTag VP64 g4+g17. Expression fold change relative to the first *fwa* plant is plotted. Error bars represent the mean ± s.e. of 2 technical replicates. (**b**) RPKM values from RNA-seq data of Col-0, T2 22aa SunTag VP64 nog-1, *fwa*, and 3 independent T2 lines of 22aa SunTag VP64 g4+g17. Error bars represent the mean ± s.e. of biological replicates: 3 for Col-0, 4 for T2 SunTag VP64 nog-1, 3 for *fwa*, 3 for T2 22aa SunTag VP64 g4+g17-1, 4 for g4+g17-2, and 4 for g4+g17-3. (**c**) Scatterplots showing the DEGs of T2 22aa SunTag VP64 g4+g17-1, g4+g17-2, and g4+g17-3 plants versus T2 22aa SunTag VP64 nog-1 plants. Gray dots represent non-DEGs and blue dots represent DEGs. The red dot with a black triangle represents the differential expression of *FWA*. Source data of Supplementary Figure 5a are provided as a Source Data file.

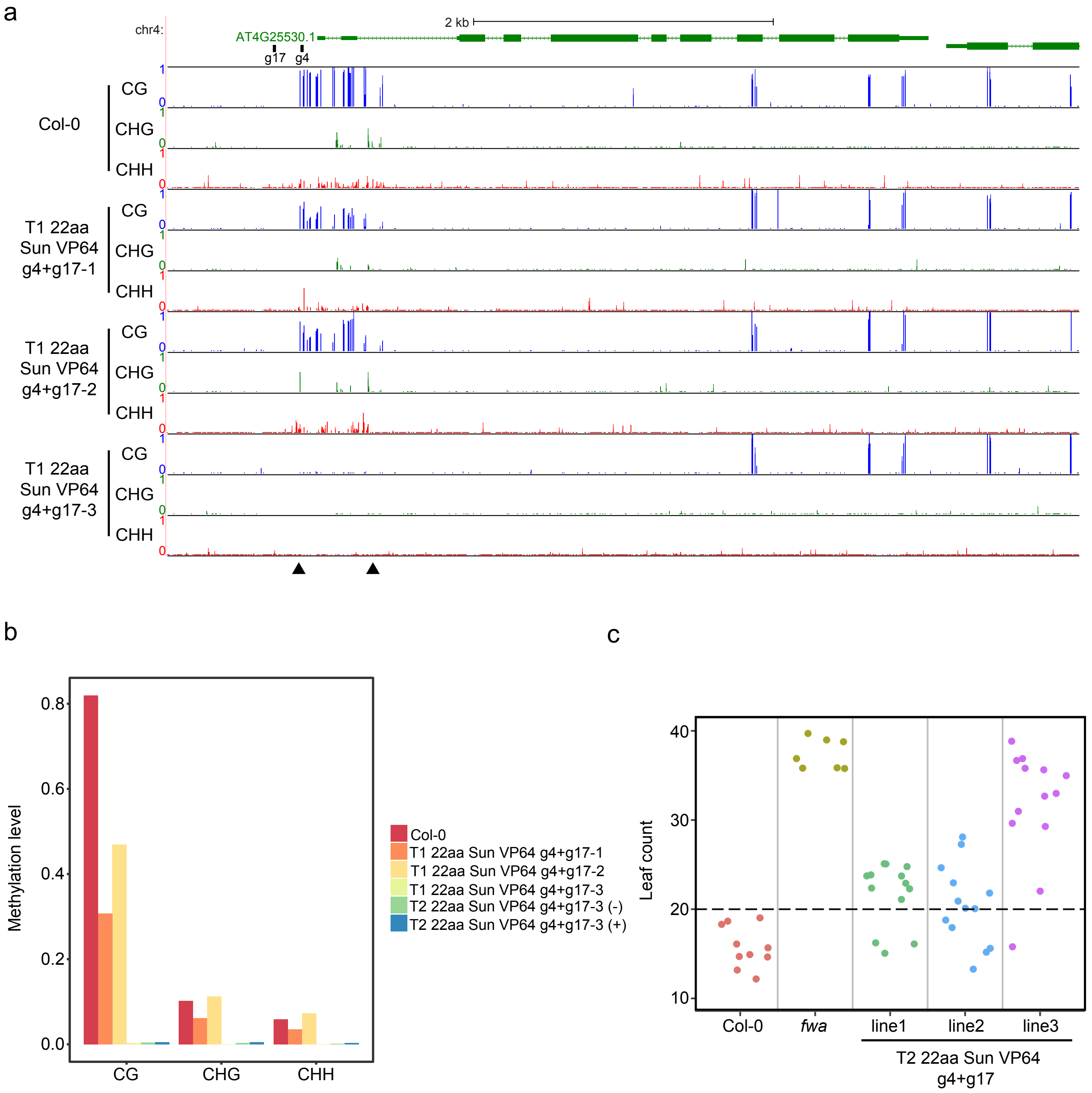

Supplementary Figure 6 *FWA* activation with g4+g17 leads to a decrease in methylation and late flowering. (**a**) WGBS tracks for all 3 sequence contexts are shown at the *FWA* genomic region for Col-0 and 3 independent T1 lines of 22aa SunTag VP64 g4+g17. The positions of gRNA17 and gRNA4 are illustrated with black bars. (**b**) Barplot showing CG, CHG, and CHH methylation levels in Col-0, 3 independent T1 lines of 22aa SunTag VP64 g4+g17, and 1 T2- and 1 T2+ plant from line 3. Black triangles in **a** indicate the boundaries used to calculate the methylation levels in the *FWA* promoter region. (**c**) A dotplot illustrating the total number of leaves of Col-0, *fwa*, and 3 independent T2 lines of 22aa SunTag VP64 g4+g17. Each dot represents one plant. Total leaf count was obtained by counting both rosette and cauline leaves. Dots above the dashed line are considered late flowering whereas those below the line are considered early flowering.

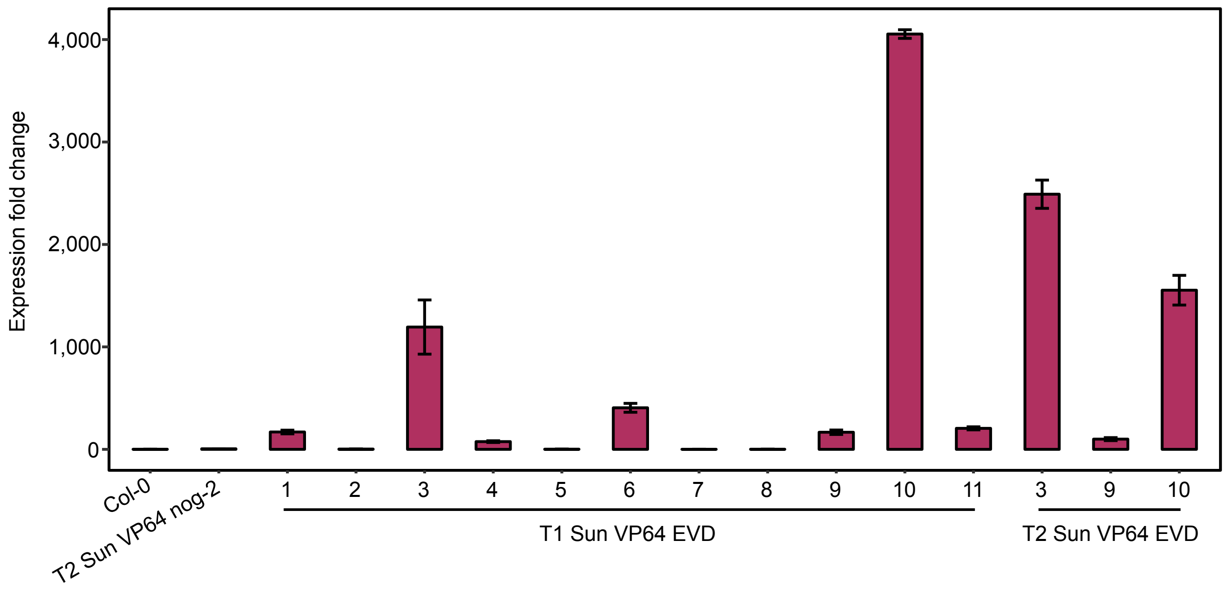

Supplementary Figure 7 SunTag VP64-mediated activation of a retrotransposon. qRT-PCR analysis of *EVD* expression levels in Col-0, T2 SunTag VP64 nog-2, 11 independent T1 lines of SunTag VP64 EVD, and 3 independent T2 lines of SunTag VP64 EVD. Expression fold change relative to Col-0 is plotted. Error bars represent the mean ± s.e. of 2 technical replicates. Source data of Supplementary Figure 7 are provided as a Source Data file.

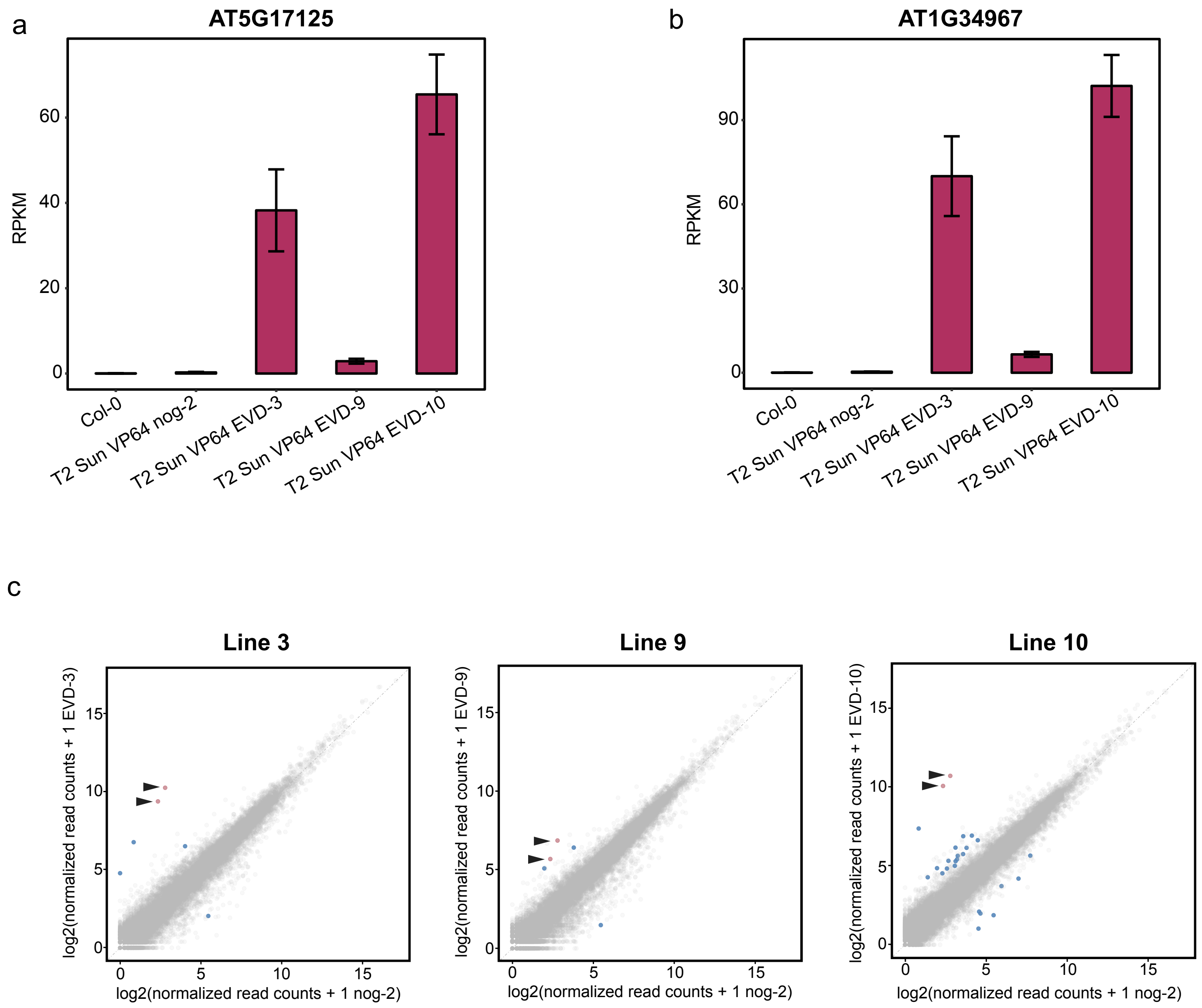

Supplementary Figure 8 Robust and specific activation of two copies of a retrotransposon. (**a**) RPKM values for *EVD* from RNA-seq data of Col-0, T2 SunTag VP64 nog-2, and 3 independent T2 lines of SunTag VP64 EVD. (**b**) RPKM values for *ATR* from RNA-seq data of Col-0, T2 SunTag VP64 nog-2, and 3 independent T2 lines of SunTag VP64 EVD. Error bars in **a** and **b** represent the mean ± s.e. of biological replicates: 3 for Col-0, T2 SunTag VP64 nog-2, T2 SunTag EVD-3, and EVD-9; and 4 for EVD-10. (**c**) Scatterplots showing the DEGs of T2 SunTag VP64 EVD-3, EVD-9, and EVD-10 plants versus SunTag VP64 nog-2 plants. Gray dots represent non-DEGs and blue dots represent DEGs. Red dots with black triangles represent the differential expression of both *EVD* and *ATR*.

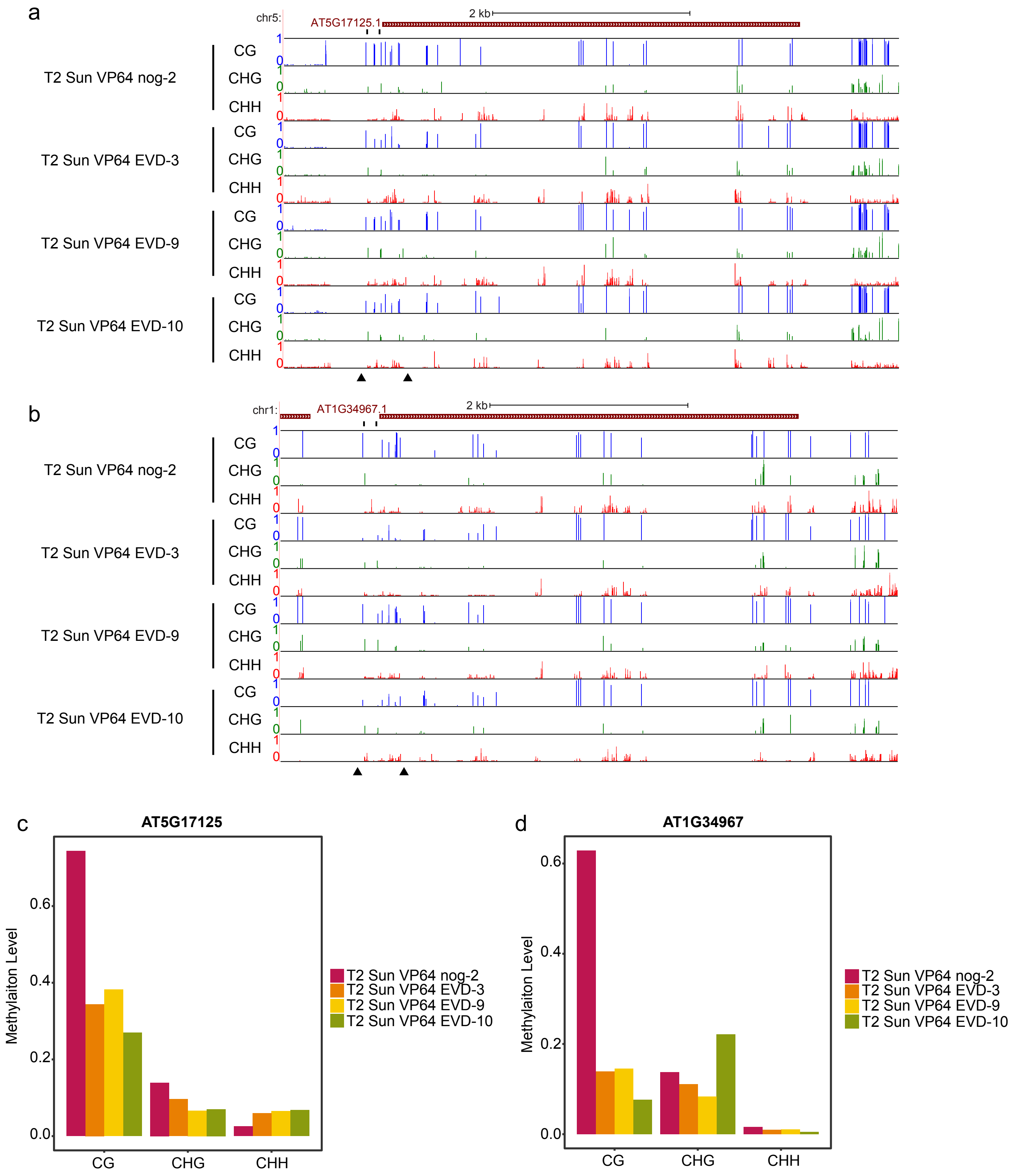

Supplementary Figure 9 Methylation levels at the *EVD* and *ATR* loci are slightly reduced upon activation. (**a**) WGBS tracks for all 3 sequence contexts are shown at the *EVD* locus for T2 SunTag VP64 nog-2 and 3 independent T2 lines of SunTag VP64 EVD. Black bars indicate the positions of the 2 gRNAs targeting the 5' end of *EVD*. (**b**) WGBS tracks for all 3 sequence contexts are shown for the *ATR* locus. (**c**) Barplot showing CG, CHG, and CHH methylation levels at the *EVD* locus for T2 SunTag VP64 nog-2 and 3 independent T2 lines of SunTag VP64 EVD. Black triangles indicate the boundaries used to calculate methylation levels. (**d**) Barplot showing CG, CHG, and CHH methylation levels at the *ATR* locus for T2 SunTag VP64 nog-2 and 3 independent T2 lines of SunTag VP64 EVD.

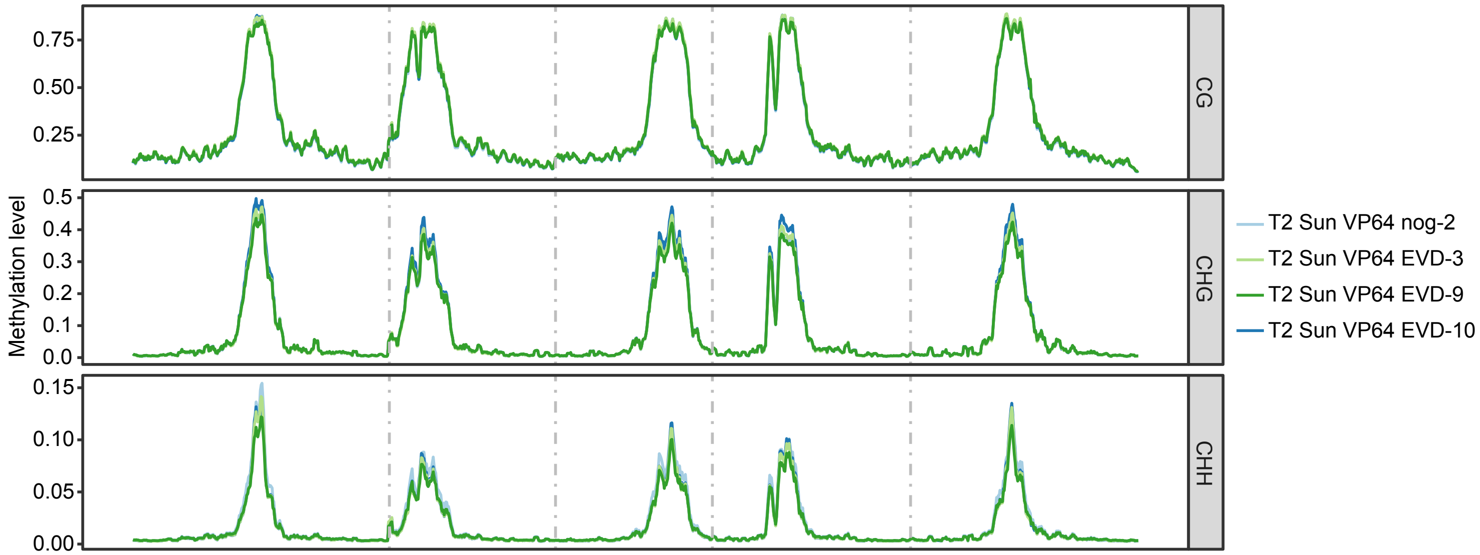

Supplementary Figure 10 Genome-wide methylation levels are unaffected in activated *EVD/ATR* lines. Chromosome-wide metaplots of CG, CHG, and CHH methylation levels of T2 SunTag VP64 nog-2 and 3 independent T2 lines of SunTag VP64 EVD. Dashed vertical lines depict the boundaries of chromosomes 1-5.

a

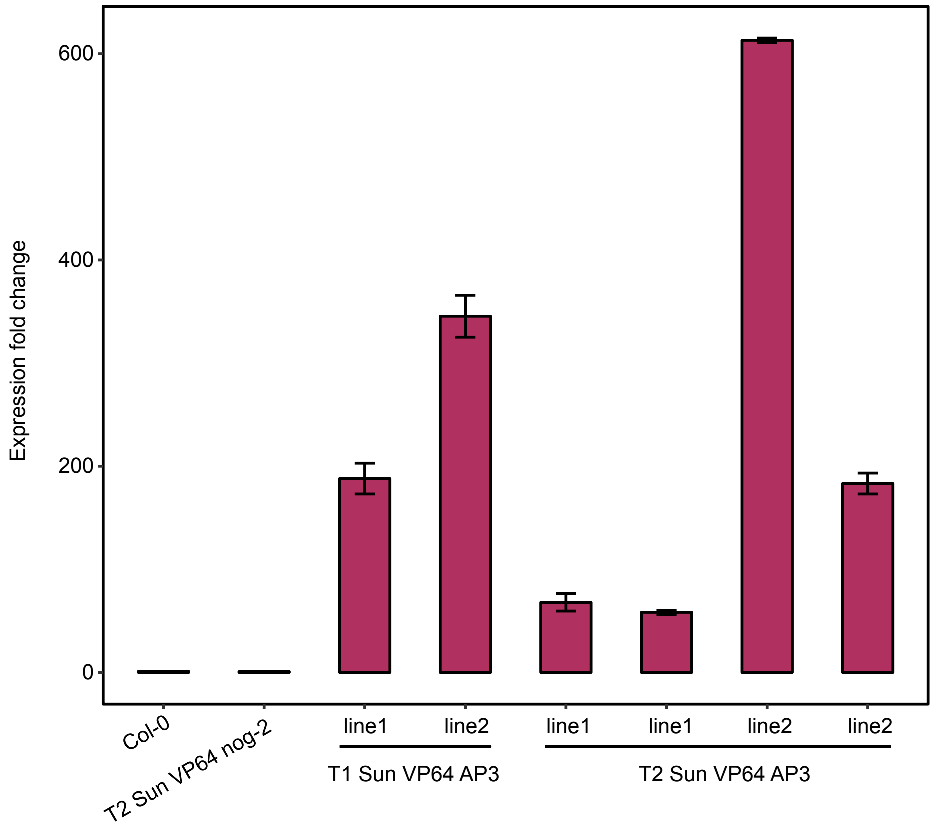

b

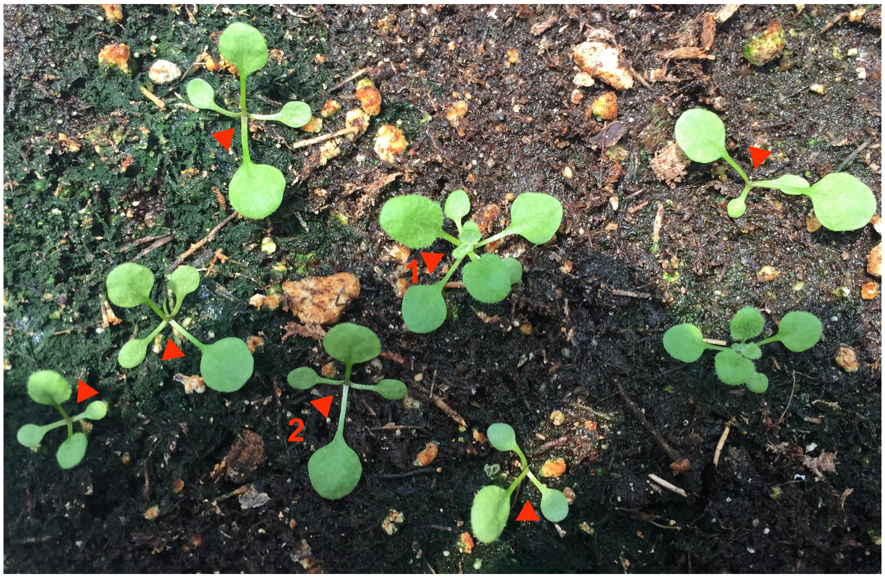

Supplementary Figure 11 SunTag VP64-mediated activation of *AP3* and *CLV3*. (**a**) qRT-PCR analysis of *AP3* expression levels in Col-0, T2 SunTag VP64 nog-2, 2 independent T1 lines of SunTag VP64 AP3, and 4 different T2 plants from 2 independent lines of SunTag VP64 AP3. Expression fold change relative to Col-0 is plotted. Error bars represent the mean ± s.e. of 2 technical replicates. (**b**) A segregating population of T2 SunTag VP64 CLV3 plants. **1**, A representative T2- plant displaying a wild type meristem. **2**, A representative T2+ plant showing a *wuschel*-like phenotype. All other plants labeled with triangles indicate other *CLV3* overexpression phenotypes. Source data of Supplementary Figure 11a are provided as a Source Data file.

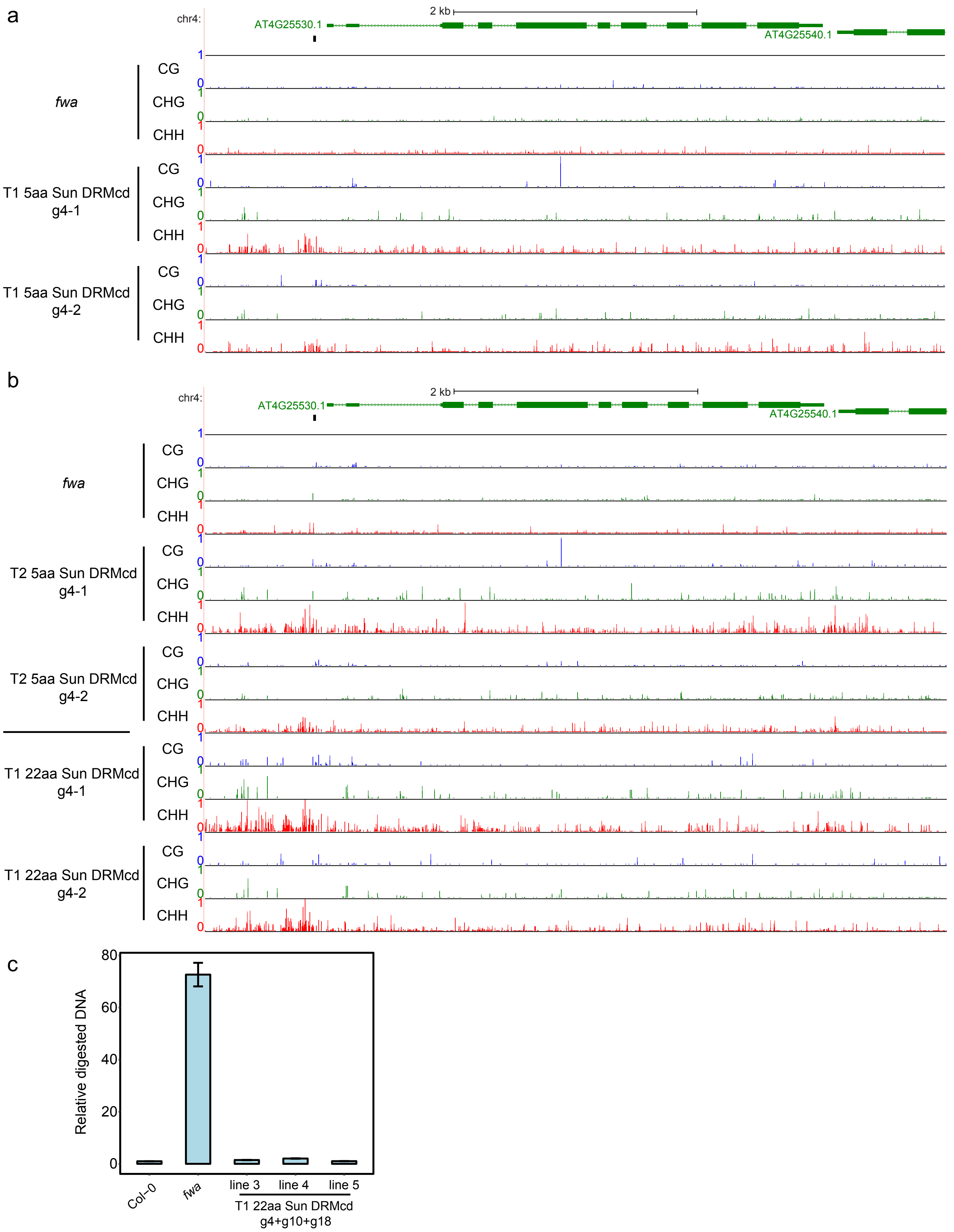

Supplementary Figure 12 The initial development of the SunTag methylation targeting system. (**a**) WGBS tracks for all 3 sequence contexts are shown at the *FWA* locus for *fwa* and 2 independent T1 lines of 5aa SunTag NtDRMcd g4. A black bar indicates the position of gRNA4. (**b**) WGBS tracks for all 3 sequence contexts are shown at the *FWA* locus for *fwa*, 2 independent T2 lines of 5aa SunTag NtDRMcd g4, and 2 independent T1 lines of 22aa SunTag NtDRMcd g4. (**c**) qPCR barplot quantifying McrBC digested DNA at the *FWA* promoter region in *fwa*, and 3 independent T1 lines of 22aa SunTag NtDRMcd g4+g10+g18 relative to a Col-0 control. Error bars represent the mean ± s.e of 2 technical replicates. Source data of Supplementary Figure 12c are provided as a Source Data file.

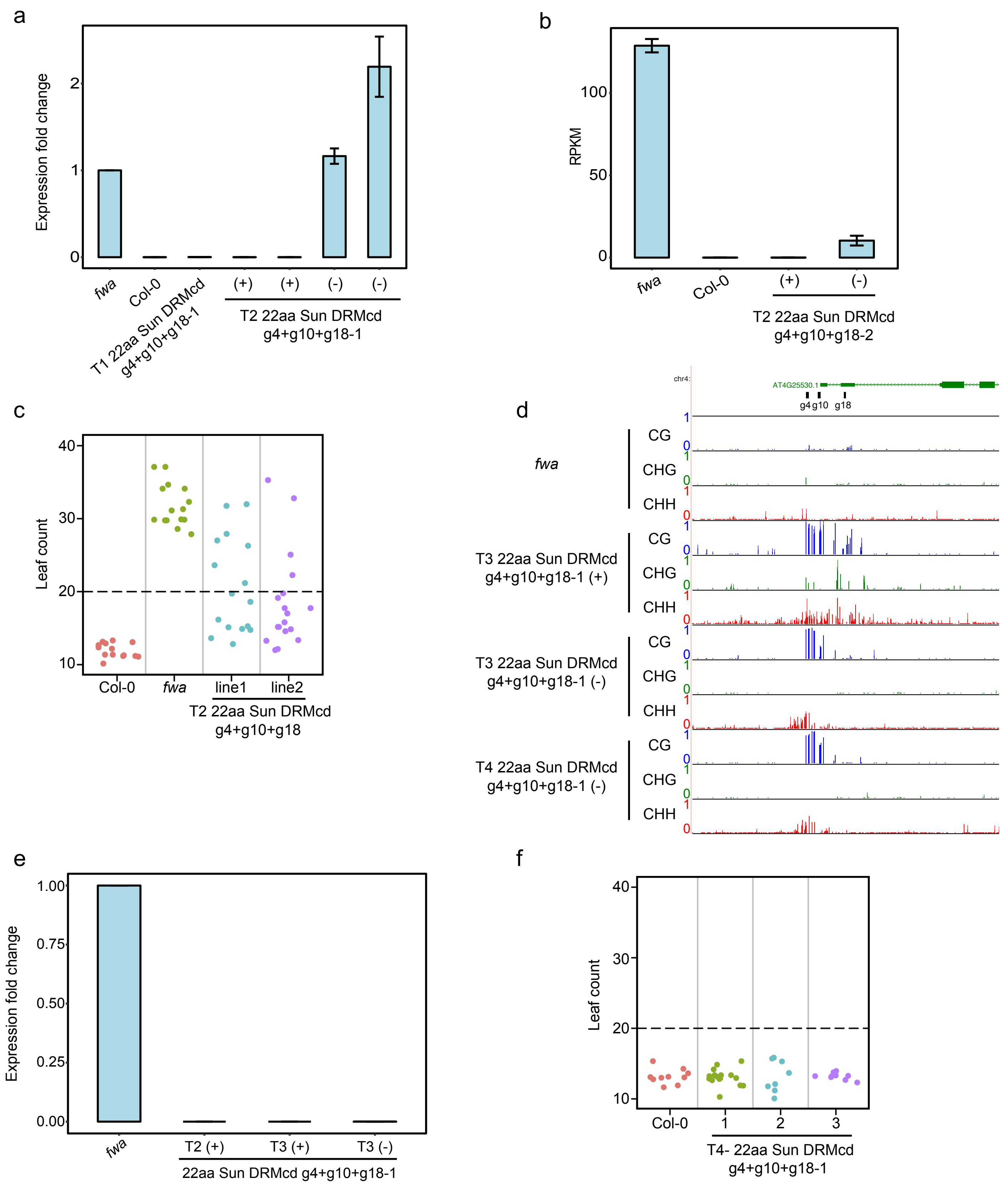

Supplementary Figure 13 NtDRMcd recruitment silences *FWA*. (**a**) qRT-PCR analysis of *FWA* expression levels in *fwa*, Col-0, T1 22aa SunTag NtDRMcd g4+g10+g18-1, and 4 different T2+ or T2- plants from 22aa SunTag NtDRMcd g4+g10+g18-1. Expression fold change relative to *fwa* is plotted. Error bars represent the mean ± s.e. of 2 technical replicates. (**b**) RPKM values from RNA-seq data of *fwa*, Col-0, and T2 22aa SunTag NtDRMcd g4+g10+g18-2 plants that had retained or segregated away the transgene. Error bars represent the mean ± s.e. of biological replicates: 3 for *fwa*, 3 for Col-0, 5 for T2+ 22aa SunTag NtDRMcd g4+g10+g18-2, and 3 for T2-. (**c**) A dotplot illustrating the total number of leaves of Col-0, *fwa*, and 2 independent T2 lines of 22aa SunTag NtDRMcd g4+g10+g18. Each dot represents one plant. Total leaf count was obtained by counting both rosette and cauline leaves. Dots above the dashed line are considered late flowering whereas those below the line are considered early flowering. (**d**) WGBS tracks for all 3 sequence contexts are shown at the *FWA* locus for *fwa*, T3+, T3-, and T4- plants from 22aa SunTag NtDRMcd g4+g10+g18-1. (**e**) qRT-PCR analysis of *FWA* expression levels in *fwa*, and T2 and T3+/- plants of 22aa SunTag NtDRMcd g4+g10+g18-1. Expression fold change relative to *fwa* is plotted. Error bars represent the mean ± s.e. of 2 technical replicates. (**f**) A dotplot illustrating the total number of leaves of Col-0 and T4- 22aa SunTag NtDRMcd g4+g10+g18-1 from 3 independent T3- populations. Source data of Supplementary Figures 13a and e are provided as a Source Data file.

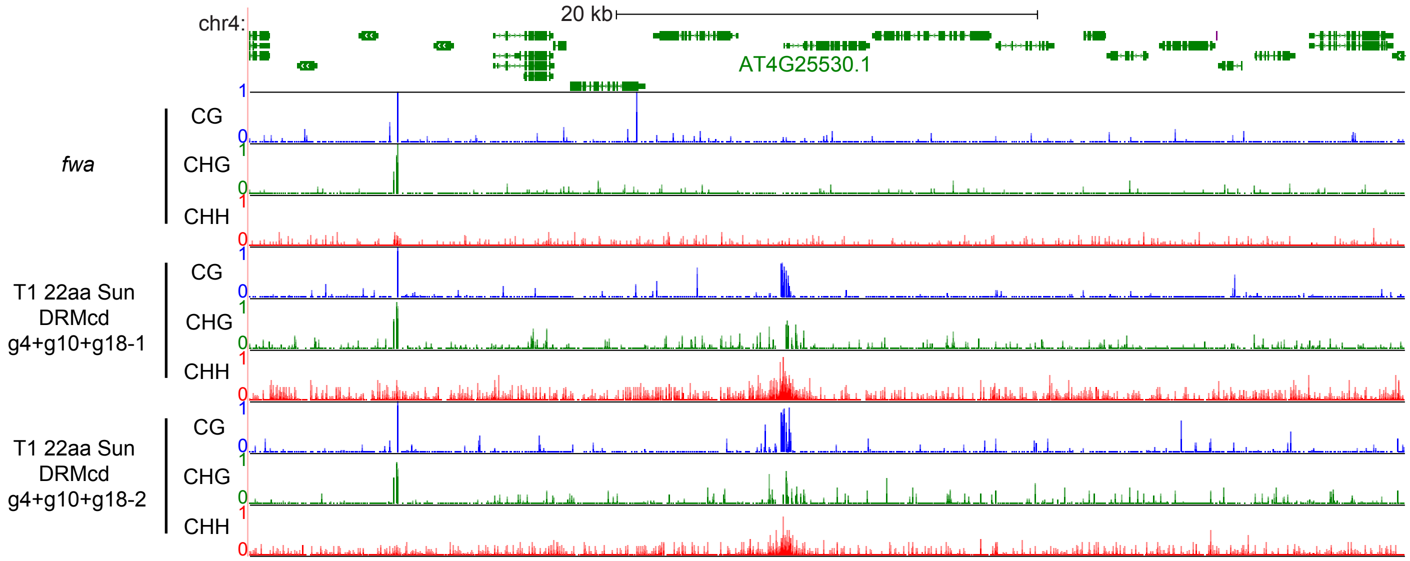

Supplementary Figure 14 NtDRMcd displays off target activity. Zoomed out WGBS tracks for all 3 sequence contexts for data shown in **Fig. 3a**.

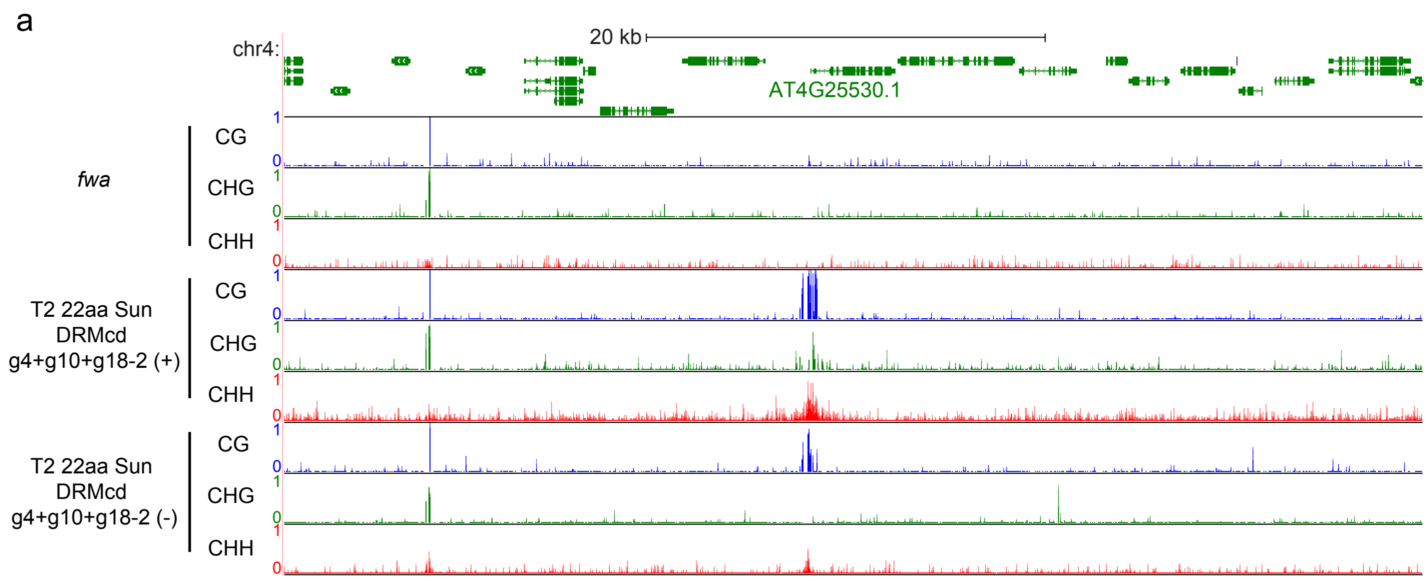

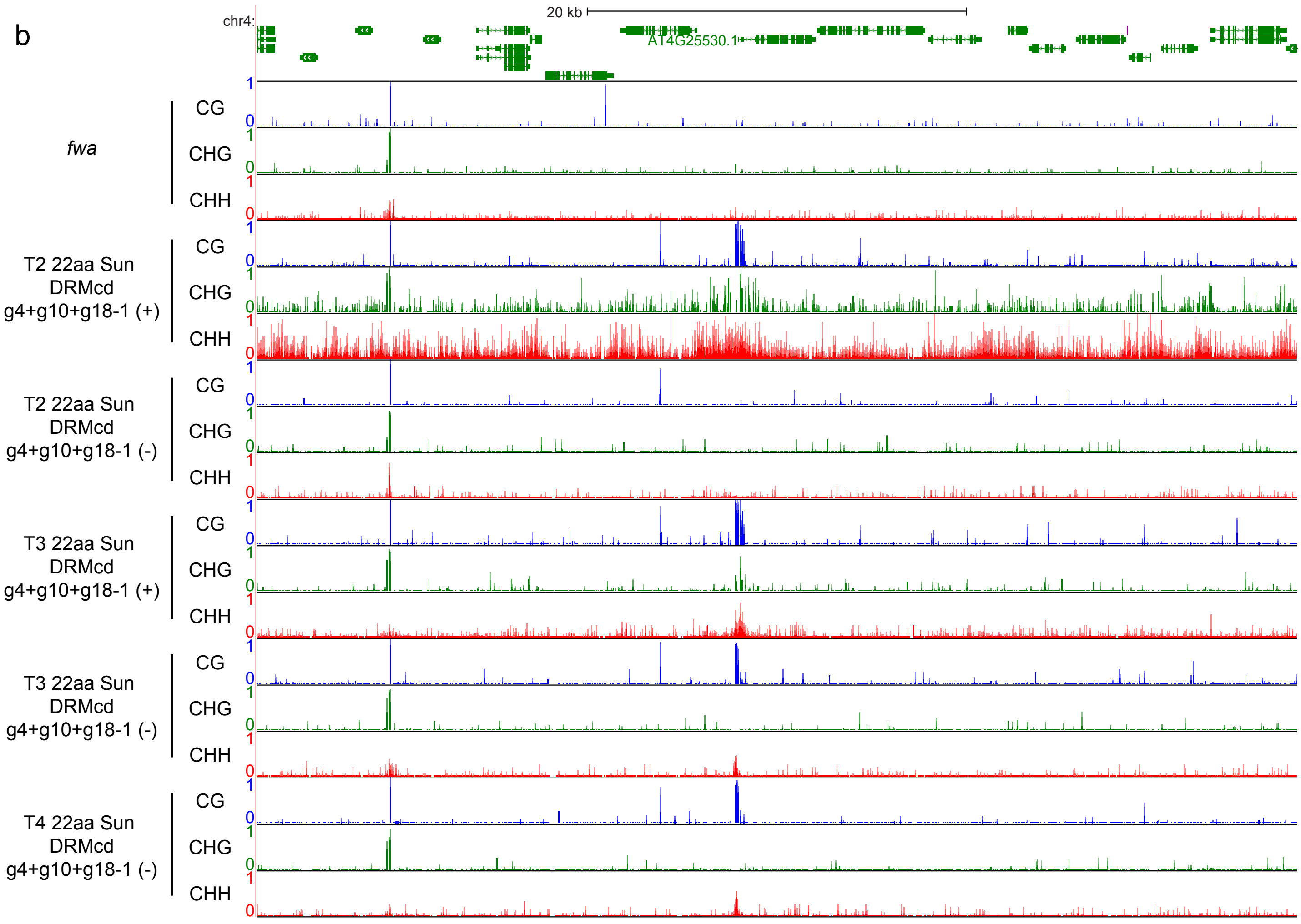

Supplementary Figure 15 Off target activity of NtDRMcd. (**a**) Zoomed out WGBS tracks for all 3 sequence contexts for data shown in **Fig. 3b**. (**b**) Zoomed out WGBS tracks for all 3 sequence contexts are shown for *fwa*, T2+ and T2- plants of 22aa SunTag NtDRMcd g4+g10+g18-1, T3+ and T3- plants, and a T4- plant from the same line.

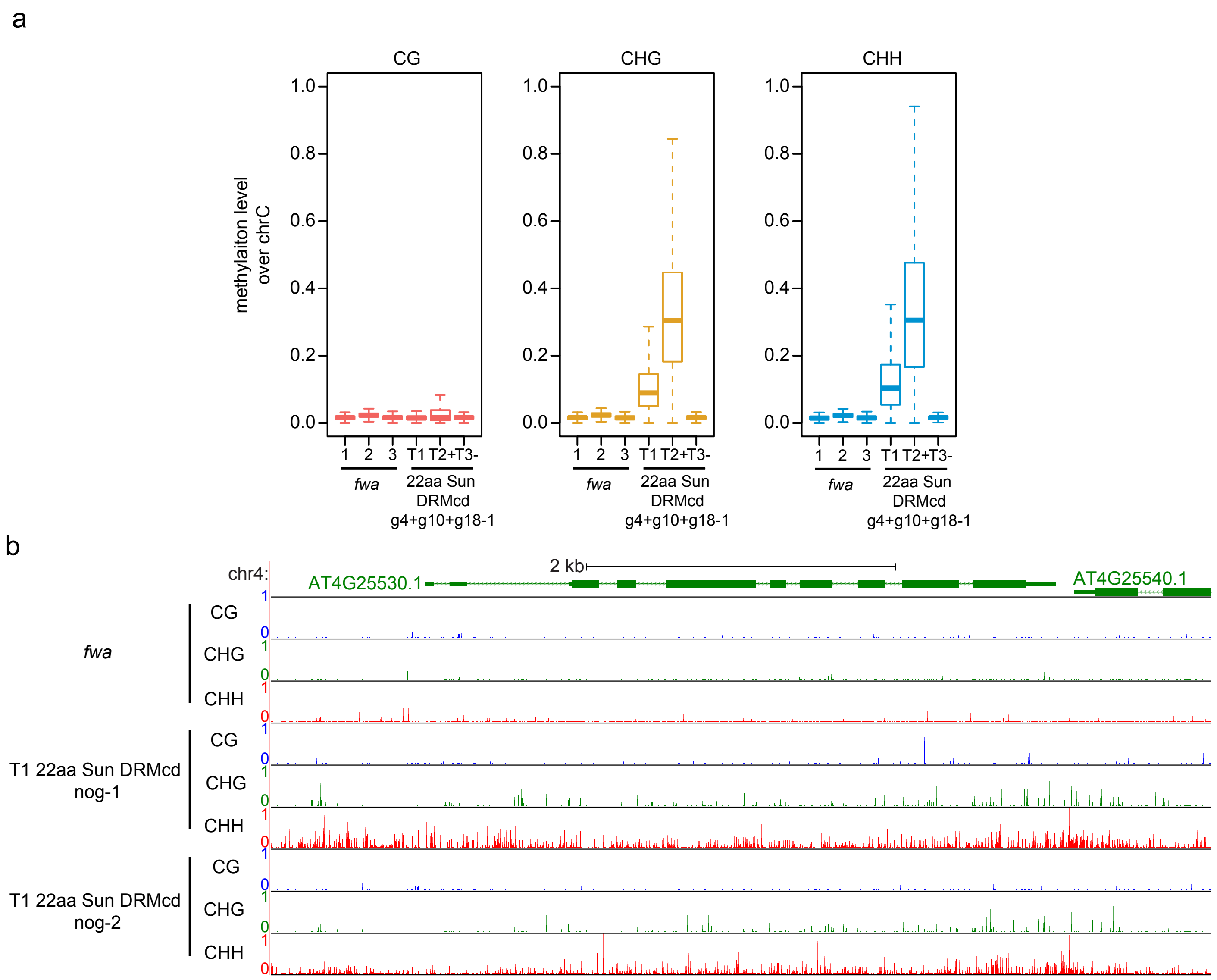

Supplementary Figure 16 scFv-sfGFP-NtDRMcd also methylates the chloroplast genome. (**a**) Boxplots showing CG, CHG, and CHH methylation levels in the chloroplast genome (chrC) for 3 *fwa* replicates and the T1, T2+, and T3- generations of 22aa SunTag NtDRMcd g4+g10+g18-1. The line indicates the median, the box limits represent the inter-quartile range (IQR), and whiskers extend to 1.5 times the IQR. (**b**) WGBS tracks for all 3 sequence contexts are shown at the *FWA* locus for *fwa* and 2 independent T1 lines of 22aa SunTag NtDRMcd nog.

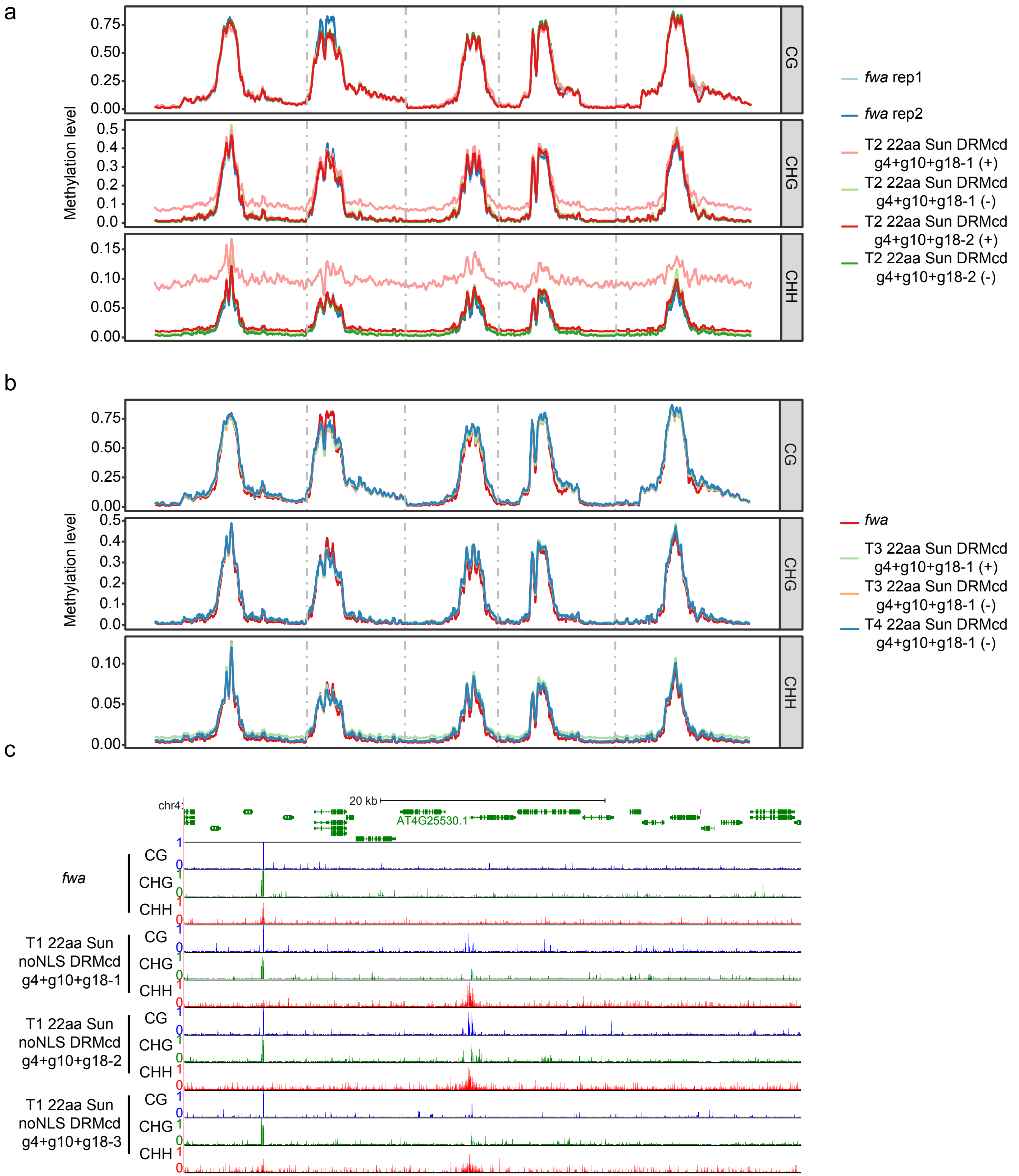

Supplementary Figure 17 Profiling the genome-wide effects of methylation targeting. (**a**) Chromosome-wide metaplots of CG, CHG, and CHH methylation levels in 2 different *fwa* plants, and 2 independent T2 lines of SunTag NtDRMcd g4+g10+g18, with T2+ and T2- plants shown for each line. (**b**) Chromosome-wide metaplots of CG, CHG, and CHH methylation levels in *fwa*, 1 T3+, 1 T3-, and 1 T4- plant of 22aa SunTag NtDRMcd g4+g10+g18-1. In all plots, dashed vertical lines depict the boundaries of chromosomes 1-5. (**c**) Zoomed out WGBS tracks for all 3 sequence contexts for data shown in **Fig. 4b**.

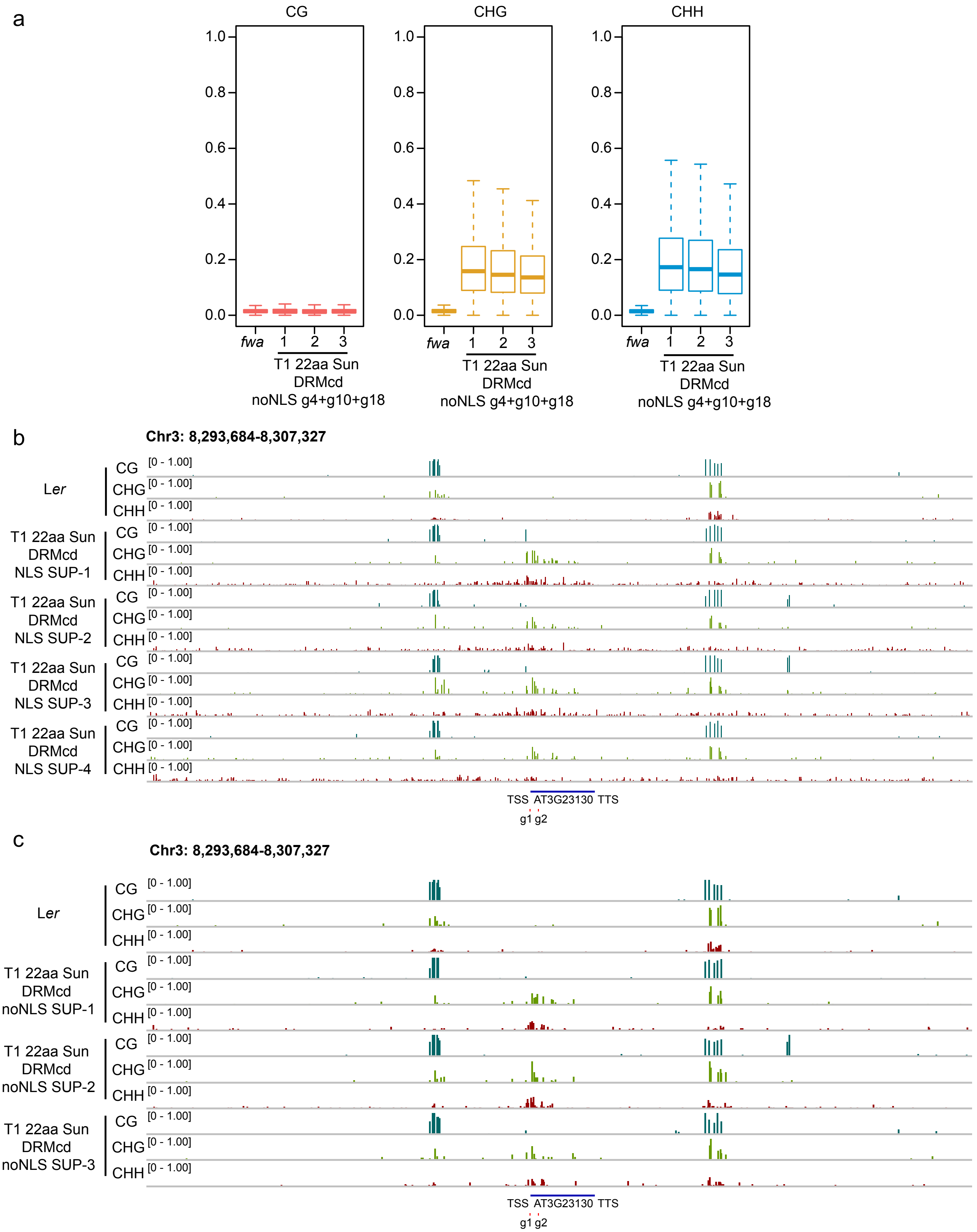

Supplementary Figure 18 SunTag NtDRMcd noNLS SUP limits off-target methylation activity of NtDRMcd. (**a**) Boxplots showing CG, CHG, and CHH methylation levels in the chloroplast genome (chrC) for *fwa* and 3 independent T1 lines of 22aa SunTag NtDRMcd noNLS g4+g10+g18. The line indicates the median, the box limits represent the inter-quartile range (IQR), and whiskers extend to 1.5 times the IQR. (**b**) Zoomed out WGBS tracks for all 3 sequence contexts for data shown in **Fig. 5a**. (**c**) Zoomed out WGBS tracks for all 3 sequence contexts for data shown in **Fig. 5b**. Red bars in **b** and **c** show both gRNA binding sites. TSS=Transcription start site and TTS=Transcription termination site.
