# Supplementary material for: Site-specific manipulation of Arabidopsis loci using CRISPR-Cas9 SunTag systems
Source: Nat Commun. 2019 Feb 13;10:729. doi: 10.1038/s41467-019-08736-7 (PMC6374409; doi:10.1038/s41467-019-08736-7)
Supplement: Supplementary file 2 — Reporting Summary [file 41467_2019_8736_MOESM2_ESM.pdf]

nature research

Corresponding author(s): Steven E. Jacobsen

Last updated by author(s): Jan 17, 2019

Reporting Summary

Nature Research wishes to improve the reproducibility of the work that we publish. This form provides structure for consistency and transparency in reporting. For further information on Nature Research policies, see Authors & Referees and the Editorial Policy Checklist.

Statistics

For all statistical analyses, confirm that the following items are present in the figure legend, table legend, main text, or Methods section.

n/a	Confirmed	
☐	☒	The exact sample size (*n*) for each experimental group/condition, given as a discrete number and unit of measurement
☐	☒	A statement on whether measurements were taken from distinct samples or whether the same sample was measured repeatedly
☐	☒	The statistical test(s) used AND whether they are one- or two-sided<br>*Only common tests should be described solely by name; describe more complex techniques in the Methods section.*
☐	☒	A description of all covariates tested
☐	☒	A description of any assumptions or corrections, such as tests of normality and adjustment for multiple comparisons
☐	☒	A full description of the statistical parameters including central tendency (e.g. means) or other basic estimates (e.g. regression coefficient) AND variation (e.g. standard deviation) or associated estimates of uncertainty (e.g. confidence intervals)
☐	☒	For null hypothesis testing, the test statistic (e.g. *F*, *t*, *r*) with confidence intervals, effect sizes, degrees of freedom and *P* value noted<br>*Give P values as exact values whenever suitable.*
☒	☐	For Bayesian analysis, information on the choice of priors and Markov chain Monte Carlo settings
☐	☒	For hierarchical and complex designs, identification of the appropriate level for tests and full reporting of outcomes
☒	☐	Estimates of effect sizes (e.g. Cohen's *d*, Pearson's *r*), indicating how they were calculated

Our web collection on statistics for biologists contains articles on many of the points above.

Software and code

Policy information about availability of computer code

Data collection	No software was used.
Data analysis	Tophat was used for aligning RNA-seq reads, R was used alongside DESeq for obtaining differentially expressed genes, Bowtie was used to align ChIP-seq reads, MACS2 was used for ChIP-seq peak calling, BS-Seeker2 was used for aligning BS-seq reads and for calling methylation, custom scripts for generating BS-seq metaplots have been deposited on GitHub

For manuscripts utilizing custom algorithms or software that are central to the research but not yet described in published literature, software must be made available to editors/reviewers. We strongly encourage code deposition in a community repository (e.g. GitHub). See the Nature Research guidelines for submitting code & software for further information.

Data

Policy information about availability of data

All manuscripts must include a data availability statement. This statement should provide the following information, where applicable:

- Accession codes, unique identifiers, or web links for publicly available datasets
- A list of figures that have associated raw data
- A description of any restrictions on data availability

High throughput sequencing data has been deposited in the Gene Expression Omnibus (GEO) database and can be accessed with the accession number GSE125230.

Field-specific reporting

Please select the one below that is the best fit for your research. If you are not sure, read the appropriate sections before making your selection.

☒ Life sciences ☐ Behavioural & social sciences ☐ Ecological, evolutionary & environmental sciences

For a reference copy of the document with all sections, see nature.com/documents/nr-reporting-summary-flat.pdf

Life sciences study design

All studies must disclose on these points even when the disclosure is negative.

Sample size	For all experiments, multiple technical and biological replicates were analyzed. In addition, multiple independent transformation events were examined for individual constructs.
Data exclusions	No data were excluded from the analyses.
Replication	Multiple independent lines, and multiple technical and biological replicates were analyzed to verify reproducibility of the results. All attempts at replication were successful.
Randomization	For the collection and analysis of plant tissue material, plants from each genotype were grown side-by-side to control for environmental differences in growing conditions.
Blinding	Because plants were grown side-by-side with appropriate controls, the investigators were not blinded.

Reporting for specific materials, systems and methods

We require information from authors about some types of materials, experimental systems and methods used in many studies. Here, indicate whether each material, system or method listed is relevant to your study. If you are not sure if a list item applies to your research, read the appropriate section before selecting a response.

Materials & experimental systems

n/a	Involved in the study	
☐	☒	Antibodies
☒	☐	Eukaryotic cell lines
☒	☐	Palaeontology
☒	☐	Animals and other organisms
☒	☐	Human research participants
☒	☐	Clinical data

Methods

n/a	Involved in the study	
☐	☒	ChIP-seq
☒	☐	Flow cytometry
☒	☐	MRI-based neuroimaging

Antibodies

Antibodies used	For ChIP, we used anti-HA.11 epitope tag antibodies (Covance, catalog #MMS-101R, clone 16B12). For western blot analysis, we used anti-HA-Peroxidase, High Affinity antibodies (Roche, catalog #12 013 819 001, clone BMG-3F10).
Validation	Validation is provided for both antibodies on the respective manufacturer websites.

ChIP-seq

Data deposition

☒ Confirm that both raw and final processed data have been deposited in a public database such as GEO.

☒ Confirm that you have deposited or provided access to graph files (e.g. BED files) for the called peaks.

Data access links<br>*May remain private before publication.*	Data has been deposited in GEO and can be accessed with the accession number GSE125230.
Files in database submission	SxaQSEQsWA148L5_idx05.fastq.gz, SxaQSEQsWA148L5_idx06.fastq.gz, summits.bed, ChIPseq_Col0.bw, ChIPseq_T2_SunTag_VP64_g4.bw, ChIPseq_T2_SunTag_VP64_g4_subtract_ChIPseq_Col0.bw
Genome browser session<br>(e.g. UCSC)	Data has been deposited in GEO and can be accessed with the accession number GSE125230.

Methodology

Replicates	A pool of Col-0 plants and a pool of plants from T2 SunTag VP64 g4 were collected for the ChIP-seq experiment.
Sequencing depth	Col-0 ChIP: 23731035 total reads, 15695534 uniquely aligned reads, single-end 50 base pair reads<br>T2 SunTag VP64 g4 ChIP: 16111240 total reads, 10402126 uniquely aligned reads, single-end 50 base pair reads
Antibodies	For ChIP, we used anti-HA.11 epitope tag antibodies (Covance, catalog #MMS-101R, clone 16B12).
Peak calling parameters	ChIP-seq analysis was done by uniquely aligning single-end 50 base pair reads to the TAIR10 genome using Bowtie, allowing two mismatches (-v 2). Subsequently, peaks were called using the MACS2 callpeak function with default parameters (q-value cutoff 0.05). Col-0 ChIP-seq was used as a control for T2 SunTag VP64 g4 ChIP-seq data.
Data quality	There are 3 peaks at FDR 5% and above 5-fold enrichment.
Software	Bowtie was used for aligning reads, SAMtools was used for removing duplicates, and MACS2 for calling ChIP-seq peaks.
